# Supplementary figures and images for: Do outcomes after kidney transplantation differ for black patients in England versus New York State? A comparative, population-cohort analysis
Source: BMJ Open. 2017 May 9;7(5):e014069. doi: 10.1136/bmjopen-2016-014069 (PMC5623361; doi:10.1136/bmjopen-2016-014069)

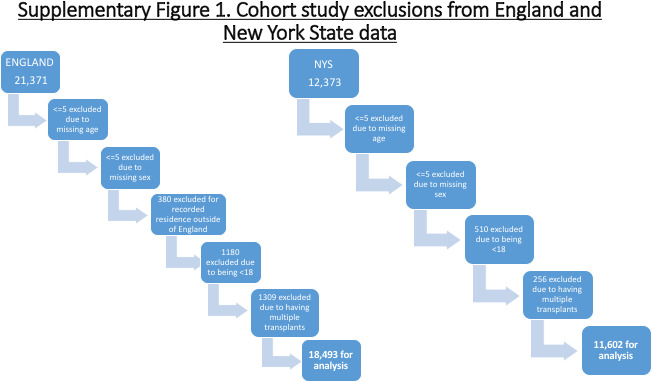

Supplement: Supplementary data [file bmjopen-2016-014069supp001.jpg]
