# Supplementary material for: Do outcomes after kidney transplantation differ for black patients in England versus New York State? A comparative, population-cohort analysis
Source: BMJ Open. 2017 May 9;7(5):e014069. doi: 10.1136/bmjopen-2016-014069 (PMC5623361; doi:10.1136/bmjopen-2016-014069)
Supplement: Supplementary data [file bmjopen-2016-014069supp003.pdf]

Supplementary Tables showing sensitivity analyses

		30 Day Mortality					
		England			NYS		
		Pre-Sensitivity Analysis	Black and Unknown combined	White and unknown combined	Pre-Sensitivity Analysis	Black and Unknown combined	White and unknown combined
Age Category	<50	1 (baseline group)	1 (baseline group)	1 (baseline group)	1 (baseline group)	1 (baseline)	1 (baseline)
	50+	3.76 (2.64,5.37)	3.76 (2.64,5.36)	3.76 (2.64,5.37)	1.21 (0.91,1.61)	1.21 (0.91,1.61)	1.21 (0.91,1.61)
Sex	Male	1 (baseline group)	1 (baseline group)	1 (baseline group)	1 (baseline group)	1 (baseline)	1 (baseline)
	Female	1.12 (0.82,1.54)	1.12 (0.82,1.54)	1.13 (0.82,1.55)	0.88 (0.66,1.17)	0.88 (0.66,1.17)	0.88 (0.66,1.17)
Admission Method	Elective	1 (baseline group)	1 (baseline group)	1 (baseline group)	1 (baseline group)	1 (baseline)	1 (baseline)
	Emergency	2.47 (1.75,3.50)	2.47 (1.74,3.49)	2.47 (1.75,3.49)	2.00 (1.49,2.68)	2.00 (1.49,2.68)	2.00 (1.49,2.68)
	Other	0.51 (0.07,3.79)	0.51 (0.07,3.73)	0.52 (0.07,3.86)	2.52 (0.59,10.71)	2.53 (0.60,10.74)	2.53 (0.60,10.75)
Number of Emergency Readmissions		0.20 (0.15,0.27)	0.20 (0.15,0.27)	0.20 (0.15,0.27)	0.77 (0.72,0.83)	0.77 (0.72,0.83)	0.77 (0.72,0.83)
Ethnic Group	Black	1 (baseline group)	1 (baseline group)	1 (baseline group)	1 (baseline group)	1 (baseline)	1 (baseline)
	Other	1.48 (0.76,2.88)	1.60 (0.97,2.63)	1.43 (0.73,2.82)	0.79 (0.55,1.15)	0.79 (0.54,1.14)	0.79 (0.54,1.15)
	White	0.81 (0.44,1.49)	0.79 (0.52,1.19)	0.73 (0.39,1.34)	0.62 (0.44,0.86)	0.61 (0.44,0.85)	0.62 (0.45,0.87)

		12 Month Mortality					
		England			NYS		
		Pre-Sensitivity Analysis	Black and Unknown combined	White and unknown combined	Pre-Sensitivity Analysis	Black and Unknown combined	White and unknown combined
Age Category	<50	1 (baseline group)	1 (baseline)	1 (baseline)	1 (baseline group)	1 (baseline)	1 (baseline)
	50+	3.97 (3.24,4.86)	3.94 (3.22,4.83)	3.97 (3.24,4.86)	1.45 (1.21,1.73)	1.21 (0.91,1.61)	1.21 (0.91,1.61)
Sex	Male	1 (baseline group)	1 (baseline)	1 (baseline)	1 (baseline group)	1 (baseline)	1 (baseline)
	Female	1.07 (0.89,1.27)	1.06 (0.89,1.27)	1.06 (0.89,1.27)	1.06 (0.89,1.26)	0.88 (0.66,1.17)	0.88 (0.66,1.17)
Admission Method	Elective	1 (baseline group)	1 (baseline)	1 (baseline)	1 (baseline group)	1 (baseline)	1 (baseline)
	Emergency	1.84 (1.53,2.22)	1.85 (1.54,2.23)	1.84 (1.52,2.22)	1.79 (1.50,2.13)	2.00 (1.49,2.68)	2.00 (1.49,2.68)
	Other	1.17 (0.59,2.33)	1.19 (0.60,2.37)	1.18 (0.59,2.33)	1.76 (0.62,4.98)	2.53 (0.60,10.74)	2.53 (0.60,10.75)
Number of Emergency Readmissions		0.84 (0.82,0.87)	0.84 (0.81,0.87)	0.84 (0.81,0.87)	0.89 (0.86,0.92)	0.77 (0.72,0.83)	0.77 (0.72,0.83)
Ethnic Group	Black	1 (baseline group)	1 (baseline)	1 (baseline)	1 (baseline group)	1 (baseline)	1 (baseline)
	Other	1.24 (0.85,1.90)	1.60 (1.17,2.18)	1.27 (0.84,1.91)	0.79 (0.63,0.99)	0.79 (0.54,1.14)	0.79 (0.54,1.15)
	White	0.94 (0.65,1.34)	1.19 (0.92,1.54)	0.91 (0.63,1.32)	0.66 (0.55,0.81)	0.61 (0.44,0.85)	0.62 (0.45,0.87)

Living and Unknown Combined

		Hazard Ratio (95% CI)	p-value
Age		1.00 (0.99,1.00)	0.181
Sex	Male	1 (baseline group)	
	Female	0.99 (0.91,1.09)	0.897
Type of Donor	Alive/unknown	1 (baseline group)	
	Dead	1.33 (1.20,1.46)	<0.001
Diabetes		1.08 (0.98,1.20)	0.12
Acute MI		0.99 (0.85,1.17)	0.945
CVF		1.03 (0.87,1.23)	0.716
PVD		1.16 (0.98,1.38)	0.086
CHF		1.16 (1.04,1.30)	0.008
Year	Pre 2007	1 (baseline group)	
	Post 2007	1.27 (1.15,1.41)	<0.001
Country	England	1 (baseline group)	
	NYS	2.17 (1.93,2.45)	<0.001

Deceased and Unknown Combined

		Hazard Ratio (95% CI)	p-value
Age		1.00 (0.99,1.00)	0.24
Sex	Male	1 (baseline group)	
	Female	1.00 (0.91,1.09)	0.96
Type of Donor	Alive	1 (baseline group)	
	Dead/Unknown	1.31 (1.16,1.47)	<0.001
Diabetes		1.09 (0.98,1.20)	0.103
Acute MI		0.99 (0.85,1.17)	0.945
CVF		1.04 (0.87,1.24)	0.655
PVD		1.17 (0.98,1.38)	0.077
CHF		1.18 (1.06,1.32)	0.004
Year	Pre 2007	1 (baseline group)	
	Post 2007	1.41 (1.27,1.56)	<0.001
Country	England	1 (baseline group)	
	NYS	2.06 (1.83,2.32)	<0.001

Alive donors only

		Hazard Ratio (95% CI)	p-value
Age		0.98 (0.97,0.99)	<0.001
Sex	Male	1 (baseline group)	
	Female	1.13 (0.92,1.39)	0.248
Diabetes		1.25 (0.98,1.58)	0.071
Acute MI		1.12 (0.74,1.70)	0.596
CVF		0.64 (0.41,1.02)	0.058
PVD		1.56 (1.05,2.31)	0.028
CHF		1.29 (0.99,1.69)	0.058
Year	Pre 2007	1 (baseline group)	
	Post 2007	1.34 (1.05,1.72)	0.02
Country	England	1 (baseline group)	
	NYS	1.68 (1.32,2.13)	<0.001

Deceased donors only

		Hazard Ratio (95% CI)	p-value
Age		1.00 (1.00,1.00)	0.915
Sex	Male	1 (baseline group)	
	Female	1.00 (0.90,1.13)	0.934
Diabetes		1.10 (0.97,1.25)	0.144
Acute MI		0.96 (0.79,1.16)	0.645
CVF		1.15 (0.94,1.41)	0.178
PVD		1.07 (0.87,1.31)	0.515
CHF		1.13 (0.99,1.30)	0.075
Year	Pre 2007	1 (baseline group)	
	Post 2007	1.25 (1.09,1.43)	0.001
Country	England	1 (baseline group)	
	NYS	2.25 (1.95,2.59)	<0.001
