# Supplementary material for: Do outcomes after kidney transplantation differ for black patients in England versus New York State? A comparative, population-cohort analysis
Source: BMJ Open. 2017 May 9;7(5):e014069. doi: 10.1136/bmjopen-2016-014069 (PMC5623361; doi:10.1136/bmjopen-2016-014069)
Supplement: Supplementary data [file bmjopen-2016-014069supp002.pdf]

Supplementary Table 1. Adjusted Cox regression analysis for transplant rejection/failure among White kidney transplant patients in England and NYS between 2003 and 2014.

		Hazard Ratio (95% CI)	p-value
Age		0.99 (0.99, 1.00)	<0.001
Sex	Male	1 (baseline group)	0.528
	Female	1.02 (0.97, 1.07)	
Type of Donor	Alive	1 (baseline group)	
	Dead	1.22 (1.26,1.40)	<0.001
	Unknown	0.99 (0.91,1.09)	0.878
Diabetes		1.17 (1.10, 1.24)	<0.001
Acute MI		1.10 (1.01, 1.20)	0.025
CVF		1.17 (1.06, 1.29)	0.002
PVD		1.14 (1.04, 1.25)	0.007
CHF		1.15 (1.06, 1.24)	0.001
Year	Pre 2007	1 (baseline group)	
	Post 2007	1.18 (1.12, 1.25)	<0.001
Country	England	1 (baseline group)	
	NYS	2.15 (2.04, 2.27)	<0.001
