# Supplementary material for: Do outcomes after kidney transplantation differ for black patients in England versus New York State? A comparative, population-cohort analysis
Source: BMJ Open. 2017 May 9;7(5):e014069. doi: 10.1136/bmjopen-2016-014069 (PMC5623361; doi:10.1136/bmjopen-2016-014069)
Supplement: Author's manuscript [file bmjopen-2016-014069.draft_revisions.pdf]

BMJ Open

Do outcomes after kidney transplantation differ for Black patients in England versus New York State? A comparative, population-cohort analysis

Journal:	*BMJ Open*
Manuscript ID	bmjopen-2016-014069
Article Type:	Research
Date Submitted by the Author:	29-Aug-2016
Complete List of Authors:	Tahir, Sanna<br>Gillott, Holly<br>Jackson-Spence, Francesca<br>Nath, Jay<br>Mytton, Jemma<br>Begaj, Irena; University Hospitals Birmingham NHS Foundation Trust, Department of Informatics<br>Sharif, Adnan
**Primary Subject Heading**:	Renal medicine
Secondary Subject Heading:	Health services research, Renal medicine, Epidemiology
Keywords:	TRANSPLANT MEDICINE, EPIDEMIOLOGY, International health services < HEALTH SERVICES ADMINISTRATION & MANAGEMENT, Quality in health care < HEALTH SERVICES ADMINISTRATION & MANAGEMENT

SCHOLARONE™
Manuscripts

For peer review only

Title. Do outcomes after kidney transplantation differ for Black patients in England versus New York State? A comparative, population-cohort analysis

Author. Sanna Tahir[1], Holly Gillott[1], Francesca Jackson Spence[1], Jay Nath[1,2], Jemma Mytton[3], Felicity Evison[3], Adnan Sharif[2,4]

Departments and Institutions.

[1]University of Birmingham, Birmingham, UK

[2]Department of Nephrology and Transplantation, Queen Elizabeth Hospital, Edgbaston, Birmingham, UK

[3]Department of Health Informatics, Queen Elizabeth Hospital, Edgbaston, Birmingham, UK

[4]School of Immunology and Inflammation, University of Birmingham, Birmingham, UK

Corresponding author contact information.

Dr. Adnan Sharif

Department of Nephrology and Transplantation

Queen Elizabeth Hospital, Edgbaston, Birmingham, B15 2WB, United Kingdom

Phone: (0121 371 5861), Fax: (0121 472 4942), Email: adnan.sharif@uhb.nhs.uk

Running title: Comparing Black post-transplant outcomes

Word count: Abstract (298), Main (3662)

For peer review only

Abbreviations

ESKD – End-stage kidney disease,

NYS – New York State

SPARCS - Statewide Planning and Research Cooperative System

HES – Hospital Episode Statistics

OPCS-4 – Classification of Interventions and Procedures version 4

ICD - International Classification of Diseases

For peer review only

For peer review only - http://bmjopen.bmj.com/site/about/guidelines.xhtml

Abstract

Objectives. While the literature suggests inferior outcomes for Black recipients of a kidney allograft in the United States, this may not be generalizable elsewhere. In this population-cohort analysis, we investigated whether kidney transplant patients of Black ethnicity have disparate outcomes in England versus New York State.

Design. A retrospective, comparative, population cohort study utilizing administrative data registries.

Settings and Participants. English data was derived from Hospital Episode Statistics, while New York State data was derived from Statewide Planning and Research Cooperative System. All adult kidney-alone allograft recipients between the years 2003 and 2013 were eligible for inclusion.

Measures. The primary outcome measure was mortality post kidney transplantation (including in-hospital death, 30-day and 1-year mortality). Secondary outcome measures included post-operative admission length of stay, risk of re-hospitalization, development of cardiac events, stroke, cancer or fracture and finally transplant rejection/failure.

Results. Black patients comprised 6.5% of the English cohort (n=1215/18,493) and 23.0% of the New York State cohort (n=2660/11,602). Compared to New York State, Black recipients of a kidney allograft in England were more likely younger, male, living-donor kidney recipients and had dissimilar medical co-morbidities. Inpatient mortality was equivalent but death within 30-days, 1-year or kidney transplant rejection/failure was lower among English versus Black patients in New York State. In adjusted regression analysis, with Black ethnicity the reference group, White patients had reduced risk for 30-day mortality (odds ratio 0.62 [95% CI 0.45-0.86], p=0.004) and 1-year mortality (odds ratio 0.66 [95% CI 0.55-0.81], p<0.001) in New York State but no difference was observed in England. Compared to

For peer review only - http://bmjopen.bmj.com/site/about/guidelines.xhtml

England, Black kidney transplant patients in New York State had increased hazard ratio for kidney transplant rejection rejection/failure by median follow up (hazard ratio 2.15, 95% CI 1.90-2.42, p<0.001).

Conclusions. Outcomes after kidney transplantation for Black patients may not be translatable between countries.

For peer review only

Article summary

Strengths and limitations of this study

- Large population-cohort study of two
- Data linkage to mortality data for accuracy
- Use of administration rather than registry data
- Kidney allograft survival data not well defined
- Numerous confounders unavailable to robustly probe this question

For peer review only - http://bmjopen.bmj.com/site/about/guidelines.xhtml

Introduction

Black patients are significantly more likely to develop end-stage kidney disease (ESKD), which is likely secondary to increased risk of chronic kidney disease progressing to kidney failure (1). Adjusted incident rates for Black patients (or African Americans) in the United States Renal Data System (USRDS) for 2010 was the highest for any racial group at 924 per million populations, compared to a rate of 276 per million populations among White patients (2). Even in countries with a lower incidence of ESKD than the United States like the United Kingdom, there is a disproportionate number of Black patients with ESKD undergoing renal replacement therapy compared to the general population (3). Therefore, strategies to optimise care for Black patients with ESKD remains of critical importance.

While kidney transplantation is recognised as the preferred modality of renal replacement therapy for patients with ESKD, disparate post-transplant outcomes are reported for Black recipients of a kidney allograft. For example, Black kidney transplant patients in the United States have previously been shown to have worse kidney allograft survival compared to White recipients (4), although recent analyses suggest reduced disparity (but still not equality) in kidney allograft outcomes between Black and White patients in the contemporary era in the United States (5). However, this is not generalizable across North America as Canadian Black transplant patients appear to not only have similar kidney allograft survival but improved mortality compared to Canadian White kidney transplant patients (6). Mortality differences after kidney transplantation between the United States and Canada are well recognised, with magnification of risk for death observed in Black Americans versus Canadians (7). In a European setting, Pallet and colleagues have shown no difference in outcomes for Black versus White patients after kidney transplantation in

For peer review only - http://bmjopen.bmj.com/site/about/guidelines.xhtml
For peer review only

France (8) and no mortality difference is observed between Black and White patients in the United Kingdom (9, 10). Therefore, inferior mortality observed for Black kidney transplant patients widely attributed to the United States may be country-specific, thus making interpretation of such data difficult for other population cohorts like the United Kingdom.

No comparative analysis has ever been conducted to specifically compare post-transplant outcomes for Black kidney transplant patients between two different countries, especially countries across distinct geographical regions like the United Kingdom and the United States. This is of interest as healthcare systems differ significantly between the two countries, with the possibility that Black people in the United Kingdom may not share the same post-transplant outcomes observed in the United States. Therefore, it is unclear if the counselling of Black kidney transplant candidates in one country can be adequately made utilizing data translated from Black patients in another country. The aim of this analysis was to undertake a comparative population-based cohort analysis exploring outcomes after kidney transplantation for Black patients in England versus New York State.

Materials and Methods

Study population

We obtained data from every kidney-alone transplant procedure performed in England and New York State between 2003 and 2013, collecting patient demographics that included age, gender, donor type (living or deceased), transplant year, medical co-morbidities (based upon ICD-10 codes) and ethnicity. English data was obtained from Hospital Episode Statistics (11), an administrative data warehouse containing admissions to all National Health Service hospitals in England. It contains detailed records relating to individual patient treatments;

For peer review only - http://bmjopen.bmj.com/site/about/guidelines.xhtml

with data extraction facilitated utilizing codes on procedural classifications (Office of Population Censuses and Surveys Classification of Interventions and Procedures, 4th revision [OPCS-4]) (12) and medical classifications (World Health Organization International Classification of Disease, 10th revision [ICD-10]) (13). The comparative analysis with the United States was performed with contemporaneous New York State data and extracted from the Statewide Planning and Research Cooperative System (SPARCS), a comprehensive all payer data reporting system collecting patient level data across New York State (14). The database collects information including patient demographics, diagnoses, procedures, and charges for every inpatient hospital admission, ambulatory surgical procedure, and emergency department admission. Individuals are assigned a unique, encrypted identification code, allowing for longitudinal analyses. Estimated reporting completeness obtained from SPARCS inpatient annual reports during the study period (2000-11) ranged from 95% to 100%, with an average of more than 98%.

This study included all kidney transplant procedures (OPCS-4 codes; M01[0-5,8,9]) performed in England and New York State between the years of 2003 to 2013. With regards to outcome analysis, both HES and SPARCS datasets have the limitation of only capturing deaths occurring in a hospital setting. To obtain the complete mortality list the study cohort was cross-referenced with mortality data from the Office for National Statistics and New York State/New York City Vital Statistics respectively, which collects information on all registered deaths in the United Kingdom and New York State respectively. Combining sources via this data linkage process creates a comprehensive dataset with regards to mortality, which was the endpoint of interest in this analysis. In addition, we extracted data for kidney transplant rejection/failure (ICD-10; T861 or ICD-9; 99681), which is derived from

For peer review only - http://bmjopen.bmj.com/site/about/guidelines.xhtml

the administrative data and substituted for lack of data linkage to transplant registry records. Formal ethical approval was not required due to the pseudo-anonymized nature of the data retrieved; data was linked by NHS Informatics utilizing a special HES ID code and avoided any patient identifiable information. Thus study was registered as an audit with University Hospitals Birmingham NHS Trust (audit identifier; CARMS-12578).

Data inclusion

We extracted data on all kidney allograft recipients between the dates of 1st January 2003 and 31st December 2013, who underwent their kidney transplant procedure in a transplant center in either England or New York State. We excluded the following patients from analysis; missing demographic data, combined solid organ transplant and pediatric cases (aged under 18 years).

Outcomes

The primary outcome measure was mortality post kidney transplantation (including in-hospital death, 30-day and 1-year mortality). In addition, we looked at various secondary outcome measures including; post-operative admission length of stay, risk of re-hospitalization, development of cardiac events, stroke, cancer or fracture and finally kidney transplant rejection/failure.

Statistical analysis

Differences between groups were compared using chi-square tests for categorical variables and Mann-Whitney tests for all continuous variables. Survival analyses were performed, where time to event data was available, using Cox's proportional hazards model and the

For peer review only - http://bmjopen.bmj.com/site/about/guidelines.xhtml

generation of Kaplan Meier plots, otherwise multivariate logistic regression models were used. The proportional hazard assumption was checked and satisfied by examination of plots of the log-negative-log of the within-group survivorship functions versus log time as well as comparing Kaplan-Meier (observed) with Cox (expected) survival curves. Variables included in the model were ethnicity, age, gender, donor type (living versus deceased), year of transplant, geography (England versus New York State) and selected medical co-morbidities (history of myocardial infarction, peripheral vascular disease, cerebrovascular disease, congestive cardiac failure and diabetes). Recipients identified as having kidney transplant rejection/failure were added as a time variate covariate in the logistic model for mortality.

Patients with missing age or gender were excluded as these variables are always used in determining the unique patient identifiers. Any missing data was assumed to be missing at random and subjected to simple listwise deletion from the analysis. Survival analyses were performed by generation of Kaplan-Meier curve estimates. A p value less than 0.05 was considered statistically significant in the analysis. All statistical analyses were conducted using Stata 14, StataCorp LP, Texas, USA.

Results

After exclusions, data was analysed for 18,493 and 11,602 patients between 2003 and 2013 from England and New York State respectively, with median follow up in England and New York State 6.3 years and 5.5 years respectively. Black patients comprised 6.5% of the English cohort (n=1215) and 23.0% of the New York State cohort (n=2660), forming the basis of all

For peer review only

comparative analyses.

Baseline demographics

Compared to Black patients in New York State, we observed significant differences in baseline demographics when compared to Black patients in England (see Table 1). Black kidney allograft recipients in England versus New York State were found to be younger, more likely to be male and more likely to get living-donor kidneys. We also observed Black recipients in England versus New York State had less medical co-morbidities listed at time of transplantation including diabetes (16.0% versus 27.9% respectively), previous myocardial infarcts (2.1% versus 3.7% respectively), strokes (1.2% versus 1.7% respectively) and congestive cardiac failure (1.8% versus 8.0% respectively), suggesting a higher level of medical co-morbidity listed at the time of kidney transplantation for Black patients in New York State versus England.

Post-transplant outcomes

There were significant differences in the post-transplantation course for Black kidney transplant patients in England versus New York State (see Table 2). Black patients in England versus New York State had longer post-operative inpatient stays and had a greater tendency to be readmitted within the first year post-transplant, but overall mean number of emergency re-hospitalisation episodes' post-transplant by median follow up was similar between the two cohorts (4.3 versus 4.0 respectively, p=0.07). With regards to medical events occurring post kidney transplantation, Black patients in England versus New York State were less likely to have cardiac events (6.0% versus 12.6% respectively, p<0.001), but

For peer review only - http://bmjopen.bmj.com/site/about/guidelines.xhtml
For peer review only

no difference was observed in episodes of cancer, strokes or fractures after kidney transplantation for Black patients in England versus New York State.

Table 2 demonstrates the difference in mortality outcomes comparing Black kidney transplant patients in England versus New York State. While inpatient mortality was equivalent comparing Black patients in England versus New York State (0.7% versus 1.4% respectively, p=0.100), mortality was significantly lower among Black patients in England versus New York State within 30-days (1.0% versus 2.3% respectively, p=0.006) and 1-year (2.8% versus 6.5% respectively, p<0.001) respectively) after kidney transplantation.

Regression analysis

While our analyses revealed significant mortality differences between Black patients in England versus New York State, we were keen to understand if Black ethnicity itself was a risk factor for mortality in each population cohort. In a logistic regression model, accounting for variables associated with mortality after kidney transplantation, we analysed whether Black ethnicity was an independent predictor for death in England or New York State. With Black ethnicity utilised as the reference group, Table 3 displays the output from the logistic regression analysis and shows White kidney allograft recipients had reduced risk for 30-day mortality (odds ratio 0.62 [95% confidence interval 0.44-0.86], p=0.005) and 1-year mortality (odds ratio 0.66 [95% confidence interval 0.55-0.81], p<0.001) in New York State but no difference was observed in England.

For peer review only

For peer review only - http://bmjopen.bmj.com/site/about/guidelines.xhtml

A significant difference was observed in unadjusted Kaplan-Meier plots of kidney transplant rejection/failure for Black kidney transplant patients in England versus New York States (see Figure 1). After adjustment for baseline variables, Cox regression analysis confirmed Black kidney transplant patients in New York State versus England had over the double the hazard ratio for kidney transplant rejection/failure by median follow up (hazard ratio 2.15, 95% CI 1.90-2.42, p<0.001) (see Table 4).

Discussion

Our comparative analysis of two large population-base cohort datasets demonstrates Black kidney transplant patients have different baseline characteristics and post-transplant outcomes in England compared to New York State. Most importantly, Black patients in England have lower mortality than their New York State counterparts, where Black ethnicity in the latter cohort was found to be independently associated with increased 30-day and 1-year mortality. Our analysis, to our knowledge, is the first comparative study comparing Black kidney allograft outcomes in contemporaneous population cohorts. Our study highlights significant differences between Black kidney transplant patients in England versus New York State and suggests caution in translating outcomes for Black transplant patients between different countries.

The strengths and weaknesses of this study must be appreciated for the correct interpretation of our results. There are likely to be numerous confounders that have an impact on Black mortality post kidney transplantation that we were unable to factor in (e.g. smoking, lifestyle, socio-cultural factors, dialysis vintage etc.). It should be acknowledged that New York State data may not be completely representative of other States in the

For peer review only - http://bmjopen.bmj.com/site/about/guidelines.xhtml

United States and we are crudely inferring that data from New York State is broadly representative of the United States. Missing data (and misclassification bias) also has an implication on the analyses performed and this limitation is inherent with all epidemiological studies of this type. Unfortunately, while the majority of data obtainable was matched between the two cohorts, we were unable to extract any information regarding socioeconomic deprivation status or cause of death from the SPARCS dataset. Our utilization of the composite for kidney transplant rejection/failure from administration records is an inferior surrogate compared to registry-derived data regarding kidney allograft rejection and graft failure. The ability to extract more data, and to link datasets to create more comprehensive databases, allows the minimization of confounding and provides a more robust platform from which to conduct meaningful epidemiological analysis. Finally, mortality data is readily available and was the sole analytical endpoint of this analysis. However, absence of recorded death may not necessarily translate to assumption that a recipient remains alive (e.g. lost to follow up due to emigration).

Current literature suggests Black kidney transplant patients in the United States have inferior kidney allograft survival compared to White recipients (4) and risk for death is magnified among Black American versus Canadian kidney transplant patients (7). Reassuringly, recently published work demonstrates significant improvement in post kidney transplant outcomes in the United States over the last two decades, more so for Black versus White patients, leading to reduced disparity (5). This may be related to advances in immunosuppression and post-transplant management, possibly benefiting Black kidney transplant patients more significantly who perhaps were more disproportionately overburdened by immunological barriers in the past. While some disparity remained in their

For peer review only - http://bmjopen.bmj.com/site/about/guidelines.xhtml

analysis in relation to significantly raised adjusted hazard ratios for Black patients and risk for graft loss in the contemporary setting, the vastly improved outcomes were encouraging for transplant clinicians to actively promote kidney transplantation among Black patients. By contrast, no mortality difference between Black and White patients has been observed in the few limited publications within European transplant centres (8-10). Our data therefore supports the inference from existing literature about disparate outcomes for Black patients in the United States versus elsewhere and raises two important questions; 1) why outcomes for Black transplant patients in the United States remain inferior to White patients, and; 2) why Black transplant patients in the United States have inferior outcomes in comparison to Black patients outside of the United States. It could be argued that the Black population in England differs from the United States, but this is an insufficient explanation as Black people in Canada and the United States share geographical origins but still have dissimilar mortality outcomes (7). It is important to distinguish the significant differences in baseline demographics between our two Black cohorts. For example, medical co-morbidities such as history of diabetes and atherosclerotic diseases were less common among Black transplant recipients in England compared to New York State, suggesting a higher level of cardiovascular burden at baseline for the latter. This could explain the significantly increased risk for cardiac events within the first year after kidney transplantation for Black kidney transplant patients in New York State versus England. Due to the absence of cause of death from the SPARCS data, we were unable to ascertain the nature of deaths among Black patients in New York State but an increase in cardiac-deaths would be a reasonable assumption. However, baseline demographics alone cannot account for the significant difference in mortality.

For peer review only - http://bmjopen.bmj.com/site/about/guidelines.xhtml
For peer review only

Perhaps the most fundamental difference between countries is the infrastructure delivering and providing healthcare. Black kidney transplant patients in England have universal health coverage as part of the taxpayer-funded National Health Service. This ensures complete financial access not just to immunosuppressive medications but also other aspects of care including allied medications (e.g. anti-hypertensive agents, glucose-lowering agents, lipid-lowering drugs, etc.) and long-term medical follow up to support post-transplant care. Compared to the United States, countries such as England, Canada and France that demonstrate equivalent mortality outcomes after transplantation for Black versus White patients share elements of universal health coverage (6, 8-10) and this is an obvious factor to explain disparate mortality outcomes. However, universal health coverage alone is unlikely to be the sole explanation for differing outcomes. Prospective clinical trials in the United States, which may attenuate the risk of any lack of access, still demonstrate poorer outcomes for Black patients in sub-group analyses (15), suggesting health coverage alone does not fully explain poor outcomes. This is supported by evidence within the United States shown by Chakkera and colleagues in their exploration of racial disparities using kidney transplant cohorts, which included patients receiving health care within versus outside the Department of Veterans Affairs (VA) (16). The VA is the largest integrated managed healthcare system in the United States and provides comprehensive medical care to eligible veterans including coverage of prescription drugs. Perhaps surprisingly, the association of Black ethnicity with poorer outcomes was consistent in non-VA versus VA users, with the latter associated with increased risk of graft loss (adjusted graft failure 1.31, 95% CI 1.10-1.57) and death (adjusted mortality 1.10, 95% CI 0.90-1.34). While noting adjusted mortality for VA users was not significantly raised in the analysis (likely due to a smaller cohort of 1,646 VA users versus 77,715 non-VA users), the adjusted risks for graft

For peer review only - http://bmjopen.bmj.com/site/about/guidelines.xhtml

failure and death were almost identical between VA and non-VA users (1.31 and 1.31 respectively for graft failure; 1.10 and 1.11 respectively for mortality).

This raises the question as to what underlies poor outcomes among Black kidney transplant patients in the United States, while acknowledging disparate outcomes appear to be narrowing between Black and White patients post kidney transplantation (5). Factors such as non-adherence, which could be a surrogate for non-affordability of drugs in the United States, is a phenomenon that is common in transplantation cohorts everywhere (including countries with universal health coverage) (17). There could be selection bias from poor access to transplantation for Black people, which has been acknowledged in transplant centers across the world, and has been comprehensively reviewed elsewhere (18). However, from the most up-to-date census reports, individuals of Black ethnicity make up 3.5% of the English population (19) and 17.6% of the New York State population (20) and Black patients formed 6.5% and 23.0% of our transplant cohorts respectively. While this suggests Black patients are over-represented as kidney allograft recipients in both England and New York State respectively, it must be remembered that ESKD is more prevalent in Black communities and therefore they are more likely to need renal replacement therapy. In should be noted that disparate post-transplant outcomes between Europe and the United States are not unique to kidney allograft recipients of Black ethnicity alone. Gondos and colleagues, analysing data from the United Network for Organ Sharing (UNOS) and the Collaborative Transplant Study (CTS), compared kidney transplant outcomes in the United States versus Europe respectively (21). Adjusted hazard ratios for graft loss were higher in the United States compared to Europe and identifying the contributing factors to this disparity should be considered a high priority to improve the delivery of post-transplant

For peer review only - http://bmjopen.bmj.com/site/about/guidelines.xhtml

care. Socioeconomic status, education and poverty are all likely to be important (22-24). Black transplant patients in the United States have an inherent risk for higher mortality or have weaker social support networks or negative health behavior that drives risk for adverse outcomes (such as smoking, alcohol consumption, sedentary lifestyle and poor diet). It is clear our understanding of the social epidemiological impact on health inequalities is limited (25), but deepening our understanding of these issues may further reduce the disparity in mortality outcomes seen in the United States for Black kidney allograft recipients.

Our analysis should be interpreted in the context of the well recognized observation of reduced risk for death for Black versus White ESKD patients after commencement of dialysis and this phenomenon has been noted in both the United States (2) and the United Kingdom (3). However, inferring that *all* Black patients on dialysis have reduced risk for death on dialysis may be incorrect when interpreting large population cohorts in the context of competing factors. For example, Kucirka and colleagues conducted an observational cohort study of 1,330,007 incident end-stage renal disease patients in the United States Renal Data System (USRDS) and showed Black patients have lower mortality on dialysis than White patients (adjusted hazard ratio 0.84; 95% confidence interval 0.83-0.84; p<0.001) (26). However, after stratification by age and treating kidney transplantation as a competing risk, Black patients below the age of 50 had significantly *higher* mortality than their White counterparts on dialysis. Teasing out the differences that help explain our disparate mortality outcomes between Black patients in England versus New York State will likely prove more challenging than just a simple explanation. Caution must also be exercised in the interpretation of epidemiological data to ensure false inferences are not made from

For peer review only - http://bmjopen.bmj.com/site/about/guidelines.xhtml

For peer review only

statistical analyses. For example, Yeates and colleagues found Black kidney transplant patients in Canada had significantly lower post-transplantation mortality compared with White patients (hazard ratio 0.49; 95% confidence interval 0.28-0.88; p=0.02) (6). This finding is surprising, and the only documented example of this observation, which requires corroboration before any legitimate interpretation can be made. However, there are recognized steps that can be taken to reduce the risk of post-transplant mortality for Black patients including greater use of living-kidney donors, which has been shown to improve outcomes and reduces racial disparity of post-transplant outcomes (27).

To conclude, this is the first comparative study examining mortality for Black kidney transplant patients between two different countries and found Black kidney transplant patients in New York State have a greater hazard for death and kidney transplant failure/rejection compared to their Black counterparts in England. Our results suggest further work is essential to investigate poor outcomes for Black patients after kidney transplantation in the United States, to aid our inadequate understanding of their social, environmental and/or biological pressures. While macro-level factors like universal health coverage may be an important factor contributing to post-transplantation mortality and allograft loss for Black patients, our study suggests additional micro-level factors are likely to be concomitantly important and are in need of deeper evaluation.

Funding

We are grateful for the funding in support of the BMedSci intercalated degrees for ST and FJS (from Kidney Research UK) and HG (from Arthur Thomson Trust).

For peer review only - http://bmjopen.bmj.com/site/about/guidelines.xhtml

Competing interests statement

We have read and understood BMJ policy on declaration of interests and declare that we have no competing interests.

Contributorship statement

Designed study[ST,JN,AS], Data extraction[ST,HG,FJS,JM,FE], Data analysis[ST,JM,FE], Data interpretation[ST,JN,AS], Wrote original draft[ST,AS], Reviewed manuscript[ST,HG,FJS,JN,JM,FE,AS]

Data sharing statement

No additional data available

References

1. Hsu CY, Lin F, Vittinghoff E, Shlipak MG. Racial Differences in the Progression from Chronic Renal Insufficiency to End-Stage Renal Disease in the United States. J Am Soc Nephrol 2003; 14: 2902-2907
2. United States Renal Data System. Chapter 1; Incidence, prevalence, patient characteristics & mortality. Accessed 5th February 2016; www.usrds.org/2012/view/v2_01.aspx
3. UK Renal Registry, 2014 – The Seventeenth Report. Accessed 5th February 2016 at; www.renalreg.org/reports/2014-seventeenth-annual-report/
4. Isaacs RB, Nock SL, Spencer CE, Connors AF Jr, Wang XQ, Sawyer R, et al. Racial disparities in renal transplant outcomes. Am J Kidney Dis 1999; 34: 706–712
5.]Purnell TS, Massie AB, Boulware LE, et al. Reduced Racial Disparity in Kidney Transplant Outcomes in the United States from 1990 to 2012. J Am Soc Nephrol 2016; published ahead of print
6. Yeates K, Wiebe N, Gill J, et al. Similar Outcomes among Black and White Renal Allograft Recipients. J Am Soc Nephrol 2009; 20: 172-179
7. Kim SJ, Schaubel DE, Fenton SSA, Leichtman AB, Port FK. Mortality After Kidney Transplantation: A Comparison Between the United States and Canada. Am J Transplant 2006; 6: 109-114
8. Pallet N, Thervet E, Alberti C, et al. Kidney transplant in black recipients: are African Europeans different from African Americans? Am J Transplant 2005; 5: 2682-7

For peer review only - http://bmjopen.bmj.com/site/about/guidelines.xhtml

9. Rudge C, Johnson RJ, Fuggle SV, et al. Renal transplantation in the United Kingdom for patients from ethnic minorities. Transplantation 2007; 83: 1169-73
10. Medcalf JF, Andrews PA, Bankart J, et al. Poorer graft survival in ethnic minorities: results from a multi-centre UK study of kidney transplant outcomes. Clin Nephrol 2011; 75: 294-301
11. Health & Social Care Information Centre: Hospital Episode Statistics online. Accessed on 6th May 2016 at; www.hscic.gov.uk/hes
12. OPCS-4 Classification – NHS Connecting for Health. Accessed on 5th February 2016; www.connectingforhealth.nhs.uk
13. World Health Organization: International Classification of Diseases (ICD). Accessed accessed 5th May 2016 at; www.who.int/classifications/icd/en
14. Statewide Planning and Research Cooperative System (SOARCS). Accessed 2nd May 2016 at; www.health.ny.gov/statistics/spacrs/
15. Woodle ES, First MR, Pirsch J, et al. A Prospective, Randomized, Double-Blind, Placebo-Controlled Multicenter Trial Comparing Early (7 Day) Corticosteroid Cessation Versus Long-Term, Low-Dose Corticosteroid Therapy. Ann Surg 2008; 248: 564-77
16. Chakkera HA, O'Hare AM, Johansen KL, et al. Influence of race on kidney transplant outcomes within and outside the Department of Veterans Affairs. J Am Soc Nephrol 2005; 16: 269-77
17. Williams AF, Manias E, Gaskin CJ, Crawford L. Medicine non-adherence in kidney transplantation. J Ren Care 2014; 40: 107-116
18. Malek SK, Keys BJ, Kumar S, Milford E, Tullius SG. Racial and ethnic disparities in kidney transplantation. Transplant Int 2010; 24: 419-424
19. Office for National Statistics 2011 Census; KS201EW Ethnic group: local authorities in England and Wales. Accessed 2nd May 2016 at; www.ons.gov.uk/ons/guide-method/census/2011
20. New York: State and County QuickFacts. Accessed 10th May 2016 at; www.quickfacts.census.gov/qfd/states/36000.html
21. Gondos A, Dohler B, Brenner H, Opelz G. Kidney Graft Survival in Europe and the United States: Strikingly Different Long-Term Outcomes. Transplantation 2013; 95: 267-274
22. Young CJ, Gaston RS. Understanding the Influence of Ethnicity on Renal Allograft Survival. Am J Transplant 2005; 5: 2603-2604
23. Goldfarb-Rumyantzev AS, Koford JK, Baird BC, Chelamcharla M, Habib AN, Wang BJ, et al. Role of socioeconomic status in kidney transplant outcome. Clin J Am Soc Nephrol 2006; 1: 313–322
24. Petersen E, Baird BC, Barenbaum LL, Leviatov A, Koford JK, Shihab F, et al. The impact of employment status on recipient and renal allograft survival. Clin Transplant 2008; 22: 428–438
25. Krieger N. Theories for social epidemiology in the 21st Century: an ecosocial perspective. Int J Epidemiol 2001; 30: 668-877
26. Kucirka LM, Grams ME, Lessler J, et al. Association of Race and Age With Survival Among Patients Undergoing Dialysis. JAMA 2011; 306: 620-626
27. Smith SR, Butterly DW. Declining influence of race on the outcome of living-donor renal transplantation. Am J Transplant 2002; 2: 282–286

For peer review only

For peer review only - http://bmjopen.bmj.com/site/about/guidelines.xhtml

Table 1. Baseline demographics of Black patients in England versus New York State

Total		England (n=1215)	NYS (n=2660)	P-value
**Age**	Mean ± standard deviation	46.3 ± 12.4	50.1 ± 13.5	<0.001
**Total Length of Stay**	Median ± interquartile range	10 ± (IQR 8-16)	6 ± (IQR 5-9)	<0.001
**Post transplant hospital stay**	Median ± interquartile range	8 ± (IQR 6-13)	6 ± (IQR 4-8)	<0.001
**Sex**	Male	743 (61.2%)	1511 (56.8%)	0.011
	Female	472 (38.8%)	1149 (43.2%)	
**Type of Donor**	Living	343 (28.2%)	548 (20.6%)	<0.001
	Deceased	856 (70.5%)	1555 (58.5%)	
	Unknown	16 (1.3%)	557 (20.9%)	
**Medical co-morbidities at time of transplantation**	Diabetes	194 (16.0%)	741 (27.9%)	<0.001
	Acute MI	25 (2.1%)	99 (3.7%)	
	Cerebrovascular disease	14 (1.2%)	46 (1.7%)	
	Congestive heart failure	22 (1.8%)	220 (8.3%)	
	Liver disease	6 (0.5%)	16 (0.6%)	
	Peptic ulcer	8 (0.7%)	31 (1.2%)	
	Peripheral vascular disease	13 (1.1%)	83 (3.1%)	

For peer review only

Table 2. Comparing 1-year and median time follow up outcomes after kidney transplantation for Black patients in England versus New York State

Variable	England	NYS	p-value
1-year follow up			
**Emergency Readmission**	782 (64.36%)	892 (33.53%)	<0.001
**Cardiac Event**	16 (1.32%)	79 (2.97%)	0.002
**Stroke**	12 (0.99%)	23 (0.86%)	<0.001
**Cancer**	17 (1.40%)	26 (0.98%)	0.245
**Fracture**	5 (0.41%)	18 (0.68%)	0.319
**Transplant Rejection/Failure**	226 (18.6%)	702 (26.4%)	<0.001
Any time Post-Transplant			
**Number of Emergency Readmissions (mean)**	4.33	3.99	0.0697
**Cardiac Event**	72 (5.93%)	336 (12.63%)	<0.001
**Stroke**	47 (3.87%)	125 (4.70%)	0.224
**Cancer**	71 (5.84%)	171 (6.43%)	0.485
**Fracture**	36 (2.96%)	84 (3.16%)	0.745
**Transplant Rejection/Failure**	367 (30.2%)	1590 (59.8%)	<0.001
Mortality			
**In Hospital Death**	9 (0.74%)	36 (1.35%)	0.099
**30 day mortality**	12 (0.99%)	61 (2.29%)	0.006
**12 month mortality**	34 (2.80%)	173 (6.50%)	<0.001

For peer review only

Table 3. Adjusted Logistic regression analysis of kidney transplant mortality in both England and New York State by ethnicity

**30-day mortality**		
ENGLAND		
**Ethnicity**	**Odds Ratio**	**p-value**
Black	1 (baseline group)	
Other	1.48 (0.76,2.88)	0.246
White	0.81 (0.44,1.49)	0.491
NEW YORK STATE		
**Ethnicity**	**Odds Ratio**	**p-value**
Black	1 (baseline group)	
Other	0.79 (0.55,1.15)	0.214
White	0.62 (0.44,0.86)	0.005
**1-year mortality**		
ENGLAND		
**Ethnicity**	**Odds Ratio**	**p-value**
Black	1 (baseline group)	
Other	1.27 (0.85,1.90)	0.234
White	0.94 (0.65, 1.34)	0.728
NEW YORK STATE		
**Ethnicity**	**Odds Ratio**	**p-value**
Black	1 (baseline group)	
Other	0.79 (0.63, 0.99)	0.064
White	0.66 (0.55,0.81)	<0.001

For peer review only

For peer review only - http://bmjopen.bmj.com/site/about/guidelines.xhtml

Table 4. Cox regression analysis for transplant rejection/failure among Black kidney transplant patients using baseline pre-transplant variables

Variable		Hazard Ratio (95% CI)	p-value
**Age**	**<50**	1 (baseline group)	
	**50+**	1.01 (0.92,1.10)	0.907
**Sex**	**Male**	1 (baseline group)	
	**Female**	1.00 (0.91,1.09)	0.946
**Type of Donor**	**Living**	1 (baseline group)	
	**Deceased**	1.35 (1.20,1.52)	<0.001
	**Unknown**	1.08 (0.92,1.27)	0.365
**Diabetes**		1.06 (0.96,1.18)	0.228
**Acute MI**		0.98 (0.83,1.15)	0.818
**Stroke**		1.03 (0.86,1.23)	0.746
**Peripheral vascular disease**		1.15 (0.97,1.37)	0.097
**Congestive heart failure**		1.16 (1.04,1.30)	0.009
**Year**	**Pre 2007**	1 (baseline group)	
	**Post 2007**	1.30 (1.16,1.45)	<0.001
**Country**	**England**	1 (baseline group)	
	**NYS**	2.14 (1.90,2.42)	<0.001

For peer review only

Figure legends

Figure 1. Cox regression plot of kidney transplant rejection/failure for Black kidney transplant patients in England versus New York State

For peer review only

For peer review only - http://bmjopen.bmj.com/site/about/guidelines.xhtml

Figure 1. Cox regression plot of kidney transplant rejection/failure for Black kidney transplant patients in England versus New York State

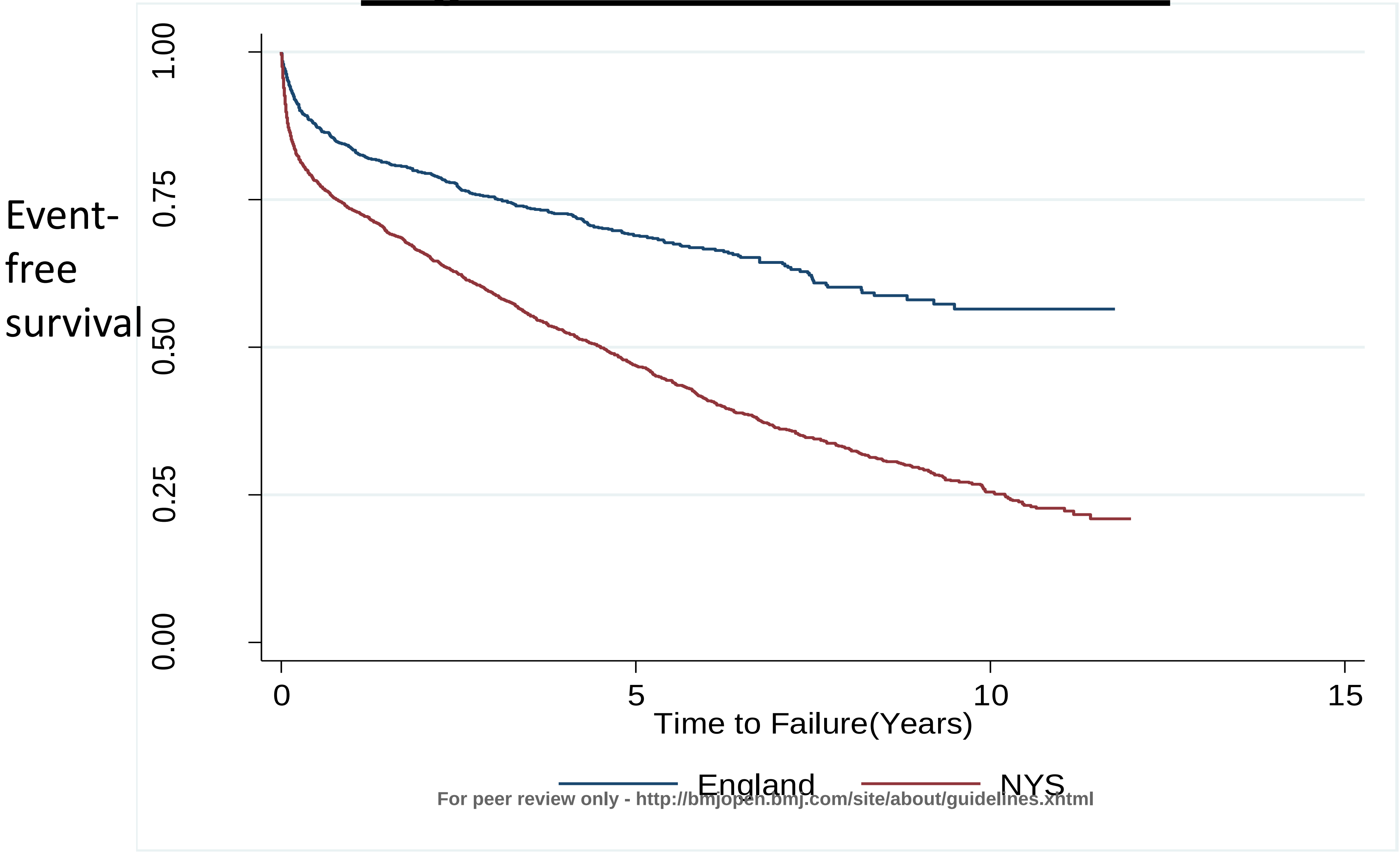

BMJ Open

Do outcomes after kidney transplantation differ for Black patients in England versus New York State? A comparative, population-cohort analysis

Journal:	*BMJ Open*
Manuscript ID	bmjopen-2016-014069.R1
Article Type:	Research
Date Submitted by the Author:	06-Jan-2017
Complete List of Authors:	Tahir, Sanna<br>Gillott, Holly<br>Jackson-Spence, Francesca<br>Nath, Jay<br>Mytton, Jemma<br>Evison, Felicity; University Hospitals Birmingham NHS Foundation Trust, Department of Informatics<br>Sharif, Adnan
**Primary Subject Heading**:	Renal medicine
Secondary Subject Heading:	Health services research, Renal medicine, Epidemiology
Keywords:	TRANSPLANT MEDICINE, EPIDEMIOLOGY, International health services < HEALTH SERVICES ADMINISTRATION & MANAGEMENT, Quality in health care < HEALTH SERVICES ADMINISTRATION & MANAGEMENT

SCHOLARONE™
Manuscripts

For peer review only

Title. Do outcomes after kidney transplantation differ for Black patients in England versus New York State? A comparative, population-cohort analysis

Author. Sanna Tahir[1], Holly Gillott[1], Francesca Jackson Spence[1], Jay Nath[1,2], Jemma Mytton[3], Felicity Evison[3], Adnan Sharif[2,4]

Departments and Institutions.

[1]University of Birmingham, Birmingham, UK

[2]Department of Nephrology and Transplantation, Queen Elizabeth Hospital, Edgbaston, Birmingham, UK

[3]Department of Health Informatics, Queen Elizabeth Hospital, Edgbaston, Birmingham, UK

[4]School of Immunology and Inflammation, University of Birmingham, Birmingham, UK

Corresponding author contact information.

Dr. Adnan Sharif

Department of Nephrology and Transplantation

Queen Elizabeth Hospital, Edgbaston, Birmingham, B15 2WB, United Kingdom

Phone: (0121 371 5861), Fax: (0121 472 4942), Email: adnan.sharif@uhb.nhs.uk

Running title: Comparing Black post-transplant outcomes

Word count: Abstract (299), Main (3856)

For peer review only

Abbreviations

ESKD – End-stage kidney disease,

NYS – New York State

SPARCS - Statewide Planning and Research Cooperative System

HES – Hospital Episode Statistics

OPCS-4 – Classification of Interventions and Procedures version 4

ICD - International Classification of Diseases

For peer review only

For peer review only - http://bmjopen.bmj.com/site/about/guidelines.xhtml

Abstract

Objectives. While the literature suggests inferior outcomes for Black recipients of a kidney allograft in the United States, this may not be generalizable elsewhere. In this population-cohort analysis, we investigated whether kidney transplant patients of Black ethnicity have disparate outcomes in England versus New York State.

Design. Retrospective, comparative, population cohort study utilizing administrative data registries.

Settings and Participants. English data was derived from Hospital Episode Statistics, while New York State data was derived from Statewide Planning and Research Cooperative System. All adult kidney-alone allograft recipients between the years 2003 and 2013 were eligible for inclusion.

Measures. The primary outcome measure was mortality post kidney transplantation (including in-hospital death, 30-day and 1-year mortality). Secondary outcome measures included post-operative admission length of stay, risk of re-hospitalization, development of cardiac events, stroke, cancer or fracture and finally transplant rejection/failure.

Results. Black patients comprised 6.5% of the English cohort (n=1215/18,493) and 23.0% of the New York State cohort (n=2660/11,602). Compared to New York State, Black recipients of a kidney allograft in England were more likely younger, male, living-donor kidney recipients and had dissimilar medical co-morbidities. Inpatient mortality was not statistically different but death within 30-days, 1-year or kidney transplant rejection/failure was lower among black patients in England versus Black patients in New York State. In adjusted regression analysis, with Black ethnicity the reference group, White patients had reduced risk for 30-day mortality (odds ratio 0.62 [95% CI 0.44-0.86]) and 1-year mortality (odds ratio 0.79 [95% CI 0.63-0.99]) in New York State but no difference was observed in England.

For peer review only

Compared to England, Black kidney transplant patients in New York State had increased hazard ratio for kidney transplant rejection rejection/failure by median follow up (hazard ratio 2.15, 95% CI 1.91-2.43).

Conclusions. Outcomes after kidney transplantation for Black patients may not be translatable between countries.

For peer review only

Article summary

Strengths and limitations of this study

- Large population-cohort study of two
- Data linkage to mortality data for accuracy
- Use of administration rather than registry data
- Kidney allograft survival data not well defined
- Numerous confounders unavailable to robustly probe this question

For peer review only - http://bmjopen.bmj.com/site/about/guidelines.xhtml

Introduction

Black patients are significantly more likely to develop end-stage kidney disease (ESKD), which is likely secondary to increased risk of chronic kidney disease progressing to kidney failure (1). Adjusted incident rates for Black patients (or African Americans) in the United States Renal Data System (USRDS) for 2010 was the highest for any racial group at 924 per million populations, compared to a rate of 276 per million populations among White patients (2). Even in countries with a lower incidence of ESKD than the United States like the United Kingdom, there is a disproportionate number of Black patients with ESKD undergoing renal replacement therapy compared to the general population (3). Therefore, strategies to optimise care for Black patients with ESKD remains of critical importance.

While kidney transplantation is recognised as the preferred modality of renal replacement therapy for patients with ESKD, disparate post-transplant outcomes are reported for Black recipients of a kidney allograft. For example, Black kidney transplant patients in the United States have previously been shown to have worse kidney allograft survival compared to White recipients (4), although recent analyses suggest reduced disparity (but still not equality) in kidney allograft outcomes between Black and White patients in the contemporary era in the United States (5). However, this is not generalizable across North America as Canadian Black transplant patients appear to not only have similar kidney allograft survival but improved mortality compared to Canadian White kidney transplant patients (6). Mortality differences after kidney transplantation between the United States and Canada are well recognised, with magnification of risk for death observed in Black Americans versus Canadians (7). In a European setting, Pallet and colleagues have shown no difference in outcomes for Black versus White patients after kidney transplantation in

For peer review only - http://bmjopen.bmj.com/site/about/guidelines.xhtml

For peer review only

France (8) and no mortality difference is observed between Black and White patients in the United Kingdom (9, 10). Therefore, inferior mortality observed for Black kidney transplant patients widely attributed to the United States may be country-specific, thus making interpretation of such data difficult for other population cohorts like the United Kingdom.

No comparative analysis has ever been conducted to specifically compare post-transplant outcomes for Black kidney transplant patients between countries across distinct geographical regions like the United Kingdom and the United States. This is of interest as healthcare systems differ significantly between the two countries, with the possibility that Black people in the United Kingdom may not share the same post-transplant outcomes observed in the United States. Therefore, it is unclear if the counselling of Black kidney transplant candidates in one country can be adequately made utilizing data translated from Black patients in another country. The aim of this analysis was to undertake a comparative population-based cohort analysis exploring outcomes after kidney transplantation for Black patients in England versus New York State.

Materials and Methods

Study population

We obtained data from every patients' first kidney-alone transplant procedure performed in England and New York State between 2003 and 2013, collecting patient demographics that included age, gender, donor type (living or deceased), transplant year, medical co-morbidities (based upon ICD-10 codes) and ethnicity. English data was obtained from Hospital Episode Statistics (11), an administrative data warehouse containing admissions to all National Health Service hospitals in England. It contains detailed records relating to

For peer review only

individual patient treatments; with data extraction facilitated utilizing codes on procedural classifications (Office of Population Censuses and Surveys Classification of Interventions and Procedures, 4th revision [OPCS-4]) (12) and medical classifications (World Health Organization International Classification of Disease, 10th revision [ICD-10]) (13). The comparative analysis with the United States was performed with contemporaneous New York State data and extracted from the Statewide Planning and Research Cooperative System (SPARCS), a comprehensive all payer data reporting system collecting patient level data across New York State (14). The database collects information including patient demographics, diagnoses, procedures, and charges for every inpatient hospital admission, ambulatory surgical procedure, and emergency department admission. Individuals are assigned a unique, encrypted identification code, allowing for longitudinal analyses. Estimated reporting completeness obtained from SPARCS inpatient annual reports during the study period (2000-11) ranged from 95% to 100%, with an average of more than 98%.

This study included all patients' first kidney transplant procedures (OPCS-4 codes; M01[0-5,8,9]) performed in England and New York State between the years of 2003 to 2013. With regards to outcome analysis, both HES and SPARCS datasets have the limitation of only capturing deaths occurring in a hospital setting. To obtain the complete mortality list the study cohort was cross-referenced with mortality data from the Office for National Statistics and New York State/New York City Vital Statistics respectively, which collects information on all registered deaths in the United Kingdom and New York State respectively. Death data was available up until December 2014. Combining sources via this data linkage process creates a comprehensive dataset with regards to mortality, which was the endpoint of interest in this analysis. In addition, we extracted data for kidney transplant rejection/failure

For peer review only - http://bmjopen.bmj.com/site/about/guidelines.xhtml

(ICD-10; T861 or ICD-9; 99681), which is derived from the administrative data and substituted for lack of data linkage to transplant registry records. Formal ethical approval was not required due to the pseudonymised nature of the data retrieved; data was linked by NHS Informatics utilizing a special HES ID code and avoided any patient identifiable information. Thus study was registered as an audit with University Hospitals Birmingham NHS Trust (audit identifier; CARMS-12578).

Data inclusion

We extracted data on all kidney allograft recipients' first procedure between the dates of 1st January 2003 and 31st December 2013, who underwent their kidney transplant procedure in a transplant center in either England or New York State. We excluded the following patients from analysis; missing age or sex data, combined solid organ transplant and pediatric cases (aged under 18 years). All patients were followed up until December 2014.

Outcomes

The primary outcome measure was all-cause mortality post kidney transplantation (including in-hospital death, 30-day and 1-year mortality). In addition, we looked at various secondary outcome measures including; post-operative admission length of stay, risk of re-hospitalization, development of cardiac events (ICD10: I2[01234] , ICD9: 410,411,413,429), stroke (ICD10: I6[0123456], ICD9: 430-436), cancer (ICD10:C00-C99, ICD9: 140-199) or fracture (ICD10: S[0123456789]2, T02, T07, T10, T12, ICD9: 800-829) and finally kidney transplant rejection/failure (ICD10: T861, ICD9: 99681). Risk of rehospitalisation was defined as whether a patient was readmitted to hospital as an emergency at any time post-

For peer review only - http://bmjopen.bmj.com/site/about/guidelines.xhtml

discharge of their transplant admission. All other secondary outcomes were captured with use of ICD10 and ICD9 codes (as defined above), at any time post-transplant.

Statistical analysis

Differences between groups were compared using chi-square tests for categorical variables two-sample t-test for normal continuous variables or Mann-Whitney tests for all non-normal continuous variables. Multivariate logistic regression was used to measure 30 day and 12 month mortality. All patients were included in these models to see if there was a difference in mortality outcomes for the different ethnic groups, within the 2 populations. Variables included in the models were age, sex, admission method (emergency v elective), number of emergency readmissions and ethnic group. Survival analyses were performed, combining all black patients in the two countries, for the outcome of graft failure/rejection (defined using ICD10 T861 or ICD 99681 at any time post-transplant) using Cox's proportional hazards model and the generation of Kaplan Meier plots. The proportional hazard assumption was checked and satisfied by examination of the scaled Schoenfeld residual plots. Variables included in the cox model were ethnicity, age, gender, donor type (living versus deceased), year of transplant, geography (England versus New York State) and selected medical co-morbidities at the time of transplant (history of myocardial infarction, peripheral vascular disease, cerebrovascular disease, congestive cardiac failure and diabetes). A *p* value less than 0.05 was considered statistically significant in the analysis. All statistical analyses were conducted using Stata 14, StataCorp LP, Texas, USA.

For peer review only - http://bmjopen.bmj.com/site/about/guidelines.xhtml

Results

Between 2003 and 2013 there were 21,371 patients in England and 12,373 patients in New York State who had their first Kidney Transplant. After exclusions due to missing age or sex, and those who had multi-organ transplants, data was analysed for 18,493 and 11,602 patients between 2003 and 2013 from England and New York State respectively, with median follow up in England and New York State 6.3 years and 5.5 years respectively. Black patients comprised 6.5% of the English cohort (n=1215) and 23.0% of the New York State cohort (n=2660), forming the basis of all comparative analyses.

Baseline demographics

Table 1 compares demographics between Black patients in England versus New York State. Black kidney allograft recipients in England versus New York State were found to be younger (46.1 versus 50.1 respectively, p<0.001), more likely to be male (61.2% versus 56.8% respectively, p=0.011) and more likely to get living-donor kidneys (28.2% versus 20.6% respectively, p<0.001). We also observed Black recipients in England versus New York State had less medical co-morbidities listed at time of transplantation including diabetes (16.0% versus 27.9% respectively, p<0.001), previous myocardial infarcts (2.1% versus 3.7% respectively, p<0.001), strokes (1.2% versus 1.7% respectively, p<0.001) and congestive cardiac failure (1.8% versus 8.0% respectively, p <0.001), suggesting a higher level of medical co-morbidity listed at the time of kidney transplantation for Black patients in New York State versus England.

Post-transplant outcomes

For peer review only - http://bmjopen.bmj.com/site/about/guidelines.xhtml

Table 2 demonstrates the difference in mortality outcomes comparing Black kidney transplant patients in England versus New York State. While inpatient mortality was similar comparing Black patients in England versus New York State (0.7% versus 1.4% respectively, p=0.099), mortality was significantly lower among Black patients in England versus New York State within 30-days (1.0% versus 2.3% respectively, p=0.006) and 1-year (2.8% versus 6.5% respectively, p<0.001)) after kidney transplantation.

There were significant differences in the post-transplantation course for Black kidney transplant patients in England versus New York State (see Tables 1 and 2). Black patients in England versus New York State had longer post-operative inpatient stays (Median 8 versus 6 days, p <0.001) and had a greater tendency to be readmitted within the first year post-transplant (64.36% versus 33.53%, p<0.001), but overall mean number of emergency re-hospitalisation episodes' post-transplant by median follow up was similar between the two cohorts (4.3 versus 4.0 respectively, p=0.07). With regards to medical events occurring post kidney transplantation, Black patients in England versus New York State were less likely to have cardiac events (6.0% versus 12.6% respectively, p<0.001), but no difference was observed in episodes of cancer, strokes or fractures after kidney transplantation for Black patients in England versus New York State.

Regression analysis

While our analyses revealed significant mortality differences between Black patients in England versus New York State, we were keen to understand if Black ethnicity itself was a risk factor for mortality in each population cohort. In a logistic regression model, accounting

For peer review only - http://bmjopen.bmj.com/site/about/guidelines.xhtml
For peer review only

for variables associated with mortality after kidney transplantation (age, sex, admission method, number of emergency readmissions), we analysed whether Black ethnicity was an independent predictor for death in England or New York State. With Black ethnicity utilised as the reference group, Table 3 displays the output from the logistic regression analysis and shows White kidney allograft recipients had reduced risk for 30-day mortality (odds ratio 0.62 [95% confidence interval 0.44-0.86]) and 1-year mortality (odds ratio 0.66 [95% confidence interval 0.55-0.81]) in New York State but no difference was observed in England (30-day mortality OR 0.81 (0.44-1.49), 1-year mortality OR 0.94 (0.65-1.34).

A significant difference was observed in unadjusted Kaplan-Meier plots of kidney transplant rejection/failure for Black kidney transplant patients in England versus New York States (see Figure 1). After adjustment for baseline variables, Cox regression analysis confirmed Black kidney transplant patients in New York State versus England had over the double the hazard ratio for kidney transplant rejection/failure during follow up (hazard ratio 2.15, 95% CI 1.91-2.43,) (see Table 4).

Discussion

Our comparative analysis of two large population-base cohort datasets demonstrates Black kidney transplant patients have different baseline characteristics and post-transplant outcomes in England compared to New York State. Most importantly, Black patients in England have lower mortality than their New York State counterparts, where Black ethnicity in the latter cohort was found to be independently associated with increased 30-day and 1-year mortality. Our analysis, to our knowledge, is the first comparative study comparing

For peer review only - http://bmjopen.bmj.com/site/about/guidelines.xhtml

Black kidney allograft outcomes in contemporaneous population cohorts. Our study highlights significant differences between Black kidney transplant patients in England versus New York State and suggests caution in translating outcomes for Black transplant patients between different countries.

The strengths and weaknesses of this study must be appreciated for the correct interpretation of our results. There are likely to be numerous confounders that have an impact on Black mortality post kidney transplantation that we were unable to factor in (e.g. smoking, lifestyle, socio-cultural factors, dialysis vintage etc.). It should be acknowledged that New York State data may not be completely representative of other States in the United States and we are crudely inferring that data from New York State is broadly representative of the United States. Missing data (and misclassification bias) also has an implication on the analyses performed and this limitation is inherent with all epidemiological studies of this type. Unfortunately, while the majority of data obtainable was matched between the two cohorts, we were unable to extract any information regarding socioeconomic deprivation status or cause of death from the SPARCS dataset. Our utilization of the composite for kidney transplant rejection/failure from administration records is an inferior surrogate compared to registry-derived data regarding kidney allograft rejection and graft failure. The ability to extract more data, and to link datasets to create more comprehensive databases, allows the minimization of confounding and provides a more robust platform from which to conduct meaningful epidemiological analysis. Further work in this area is clearly warranted and should be undertaken using more robust renal data including registry data from both the United States (e.g. US Renal Data System, Scientific registry of Transplant Recipients) and the United Kingdom (e.g. UK Transplant

For peer review only - http://bmjopen.bmj.com/site/about/guidelines.xhtml

Registry, Renal Registry). Finally, mortality data is readily available and was the sole analytical endpoint of this analysis. However, absence of recorded death may not necessarily translate to assumption that a recipient remains alive (e.g. lost to follow up due to emigration).

Current literature suggests Black kidney transplant patients in the United States have inferior kidney allograft survival compared to White recipients (4) and risk for death is magnified among Black American versus Canadian kidney transplant patients (7). Reassuringly, recently published work demonstrates significant improvement in post kidney transplant outcomes in the United States over the last two decades, more so for Black versus White patients, leading to reduced disparity (5). This may be related to advances in immunosuppression and post-transplant management, possibly benefiting Black kidney transplant patients more significantly who perhaps were more disproportionately overburdened by immunological barriers in the past. While some disparity remained in their analysis in relation to significantly raised adjusted hazard ratios for Black patients and risk for graft loss in the contemporary setting, the vastly improved outcomes were encouraging for transplant clinicians to actively promote kidney transplantation among Black patients. By contrast, no mortality difference between Black and White patients has been observed in the few limited publications within European transplant centres (8-10). Our data therefore supports the inference from existing literature about disparate outcomes for Black patients in the United States versus elsewhere and raises two important questions; 1) why outcomes for Black transplant patients in the United States remain inferior to White patients, and; 2) why Black transplant patients in the United States have inferior outcomes in comparison to Black patients outside of the United States. It could be argued that the Black population in

For peer review only - http://bmjopen.bmj.com/site/about/guidelines.xhtml

For peer review only

England differs from the United States, but this is an insufficient explanation as Black people in Canada and the United States share geographical origins but still have dissimilar mortality outcomes (7). It is important to distinguish the significant differences in baseline demographics between our two Black cohorts. For example, medical co-morbidities such as history of diabetes and atherosclerotic diseases were less common among Black transplant recipients in England compared to New York State, suggesting a higher level of cardiovascular burden at baseline for the latter. This could explain the significantly increased risk for cardiac events within the first year after kidney transplantation for Black kidney transplant patients in New York State versus England. Due to the absence of cause of death from the SPARCS data, we were unable to ascertain the nature of deaths among Black patients in New York State but an increase in cardiac-deaths would be a reasonable assumption. However, baseline demographics alone cannot account for the significant difference in mortality.

Perhaps the most fundamental difference between countries is the infrastructure delivering and providing healthcare. Black kidney transplant patients in England have universal health coverage as part of the taxpayer-funded National Health Service. This ensures complete financial access not just to immunosuppressive medications but also other aspects of care including allied medications (e.g. anti-hypertensive agents, glucose-lowering agents, lipid-lowering drugs, etc.) and long-term medical follow up to support post-transplant care. Compared to the United States, countries such as England, Canada and France that demonstrate similar mortality outcomes after transplantation for Black versus White patients share elements of universal health coverage (6, 8-10) and this is an obvious factor to explain disparate mortality outcomes. However, universal health coverage alone is

For peer review only

unlikely to be the sole explanation for differing outcomes. Prospective clinical trials in the United States, which may attenuate the risk of any lack of access, still demonstrate poorer outcomes for Black patients in sub-group analyses (15), suggesting health coverage alone does not fully explain poor outcomes. This is supported by evidence within the United States shown by Chakkera and colleagues in their exploration of racial disparities using kidney transplant cohorts, which included patients receiving health care within versus outside the Department of Veterans Affairs (VA) (16). The VA is the largest integrated managed healthcare system in the United States and provides comprehensive medical care to eligible veterans including coverage of prescription drugs. Perhaps surprisingly, the association of Black ethnicity with poorer outcomes was consistent in non-VA versus VA users, with the latter associated with increased risk of graft loss (adjusted graft failure 1.31, 95% CI 1.10-1.57) and death (adjusted mortality 1.10, 95% CI 0.90-1.34). While noting adjusted mortality for VA users was not significantly raised in the analysis (likely due to a smaller cohort of 1,646 VA users versus 77,715 non-VA users), the adjusted risks for graft failure and death were almost identical between VA and non-VA users (1.31 and 1.31 respectively for graft failure; 1.10 and 1.11 respectively for mortality).

This raises the question as to what underlies poor outcomes among Black kidney transplant patients in the United States, while acknowledging disparate outcomes appear to be narrowing between Black and White patients post kidney transplantation (5). Factors such as non-adherence, which could be a surrogate for non-affordability of drugs in the United States, is a phenomenon that is common in transplantation cohorts everywhere (including countries with universal health coverage) (17). There could be selection bias from poor access to transplantation for Black people, which has been acknowledged in transplant

For peer review only - http://bmjopen.bmj.com/site/about/guidelines.xhtml

centers across the world, and has been comprehensively reviewed elsewhere (18). However, from the most up-to-date census reports, individuals of Black ethnicity make up 3.5% of the English population (19) and 17.6% of the New York State population (20) and Black patients formed 6.5% and 23.0% of our transplant cohorts respectively. While this suggests Black patients are over-represented as kidney allograft recipients in both England and New York State respectively, it must be remembered that ESKD is more prevalent in Black communities and therefore they are more likely to need renal replacement therapy. In should be noted that disparate post-transplant outcomes between Europe and the United States are not unique to kidney allograft recipients of Black ethnicity alone. Gondos and colleagues, analysing data from the United Network for Organ Sharing (UNOS) and the Collaborative Transplant Study (CTS), compared kidney transplant outcomes in the United States versus Europe respectively (21). Adjusted hazard ratios for graft loss were higher in the United States compared to Europe and identifying the contributing factors to this disparity should be considered a high priority to improve the delivery of post-transplant care. Socioeconomic status, education and poverty are all likely to be important and Black transplant patients in the United States may an inherent risk for higher mortality or have weaker social support networks or negative health behavior that drives risk for adverse outcomes (such as smoking, alcohol consumption, sedentary lifestyle and poor diet) (22-24). It is clear our understanding of the social epidemiological impact on health inequalities is limited (25), but deepening our understanding of these issues may further reduce the disparity in mortality outcomes seen in the United States for Black kidney allograft recipients.

Our analysis should be interpreted in the context of the well recognized observation of

For peer review only - http://bmjopen.bmj.com/site/about/guidelines.xhtml

reduced risk for death for Black versus White ESKD patients after commencement of dialysis and this phenomenon has been noted in both the United States (2) and the United Kingdom (3). However, inferring that *all* Black patients on dialysis have reduced risk for death on dialysis may be incorrect when interpreting large population cohorts in the context of competing factors. For example, Kucirka and colleagues conducted an observational cohort study of 1,330,007 incident end-stage renal disease patients in the United States Renal Data System (USRDS) and showed Black patients have lower mortality on dialysis than White patients (adjusted hazard ratio 0.84; 95% confidence interval 0.83-0.84; p<0.001) (26). However, after stratification by age and treating kidney transplantation as a competing risk, Black patients below the age of 50 had significantly *higher* mortality than their White counterparts on dialysis. Teasing out the differences that help explain our disparate mortality outcomes between Black patients in England versus New York State will likely prove more challenging than just a simple explanation. Caution must also be exercised in the interpretation of epidemiological data to ensure false inferences are not made from statistical analyses. For example, Yeates and colleagues found Black kidney transplant patients in Canada had significantly lower post-transplantation mortality compared with White patients (hazard ratio 0.49; 95% confidence interval 0.28-0.88; p=0.02) (6). This finding is surprising, and the only documented example of this observation, which requires corroboration before any legitimate interpretation can be made. However, there are recognized steps that can be taken to reduce the risk of post-transplant mortality for Black patients including greater use of living-kidney donors, which has been shown to improve outcomes and reduces racial disparity of post-transplant outcomes (27).

For peer review only - http://bmjopen.bmj.com/site/about/guidelines.xhtml

To conclude, this is the first comparative study examining mortality for Black kidney transplant patients between two different countries and found Black kidney transplant patients in New York State have a greater hazard for death and kidney transplant failure/rejection compared to their Black counterparts in England. Our results suggest further work is essential to investigate poor outcomes for Black patients after kidney transplantation in the United States, to aid our inadequate understanding of their social, environmental and/or biological pressures. While macro-level factors like universal health coverage may be an important factor contributing to post-transplantation mortality and allograft loss for Black patients, our study suggests additional micro-level factors are likely to be concomitantly important and are in need of deeper evaluation.

Funding

We are grateful for the funding in support of the BMedSci intercalated degrees for ST and FJS (from Kidney Research UK) and HG (from Arthur Thomson Trust).

Competing interests statement

We have read and understood BMJ policy on declaration of interests and declare that we have no competing interests.

Contributorship statement

Designed study[ST,JN,AS], Data extraction[ST,HG,FJS,JM,FE], Data analysis[ST,JM,FE], Data interpretation[ST,JN,AS], Wrote original draft[ST,AS], Reviewed manuscript[ST,HG,FJS,JN,JM,FE,AS]

For peer review only - http://bmjopen.bmj.com/site/about/guidelines.xhtml

Data sharing statement

No additional data available

References

1. Hsu CY, Lin F, Vittinghoff E, Shlipak MG. Racial Differences in the Progression from Chronic Renal Insufficiency to End-Stage Renal Disease in the United States. J Am Soc Nephrol 2003; 14: 2902-2907
2. United States Renal Data System. Chapter 1; Incidence, prevalence, patient characteristics & mortality. Accessed 5th February 2016; www.usrds.org/2012/view/v2_01.aspx
3. UK Renal Registry, 2014 – The Seventeenth Report. Accessed 5th February 2016 at; www.renalreg.org/reports/2014-seventeenth-annual-report/
4. Isaacs RB, Nock SL, Spencer CE, Connors AF Jr, Wang XQ, Sawyer R, et al. Racial disparities in renal transplant outcomes. Am J Kidney Dis 1999; 34: 706–712
5.]Purnell TS, Massie AB, Boulware LE, et al. Reduced Racial Disparity in Kidney Transplant Outcomes in the United States from 1990 to 2012. J Am Soc Nephrol 2016; published ahead of print
6. Yeates K, Wiebe N, Gill J, et al. Similar Outcomes among Black and White Renal Allograft Recipients. J Am Soc Nephrol 2009; 20: 172-179
7. Kim SJ, Schaubel DE, Fenton SSA, Leichtman AB, Port FK. Mortality After Kidney Transplantation: A Comparison Between the United States and Canada. Am J Transplant 2006; 6: 109-114
8. Pallet N, Thervet E, Alberti C, et al. Kidney transplant in black recipients: are African Europeans different from African Americans? Am J Transplant 2005; 5: 2682-7
9. Rudge C, Johnson RJ, Fuggle SV, et al. Renal transplantation in the United Kingdom for patients from ethnic minorities. Transplantation 2007; 83: 1169-73
10. Medcalf JF, Andrews PA, Bankart J, et al. Poorer graft survival in ethnic minorities: results from a multi-centre UK study of kidney transplant outcomes. Clin Nephrol 2011; 75: 294-301
11. Health & Social Care Information Centre: Hospital Episode Statistics online. Accessed on 6th May 2016 at; www.hscic.gov.uk/hes
12. OPCS-4 Classification – NHS Connecting for Health. Accessed on 5th February 2016; www.connectingforhealth.nhs.uk
13. World Health Organization: International Classification of Diseases (ICD). Accessed accessed 5th May 2016 at; www.who.int/classifications/icd/en
14. Statewide Planning and Research Cooperative System (SOARCS). Accessed 2nd May 2016 at; www.health.ny.gov/statistics/spacrs/
15. Woodle ES, First MR, Pirsch J, et al. A Prospective, Randomized, Double-Blind, Placebo-Controlled Multicenter Trial Comparing Early (7 Day) Corticosteroid Cessation Versus Long-Term, Low-Dose Corticosteroid Therapy. Ann Surg 2008; 248: 564-77
16. Chakkera HA, O'Hare AM, Johansen KL, et al. Influence of race on kidney transplant

For peer review only

outcomes within and outside the Department of Veterans Affairs. J Am Soc Nephrol 2005; 16: 269-77
17. Williams AF, Manias E, Gaskin CJ, Crawford L. Medicine non-adherence in kidney transplantation. J Ren Care 2014; 40: 107-116
18. Malek SK, Keys BJ, Kumar S, Milford E, Tullius SG. Racial and ethnic disparities in kidney transplantation. Transplant Int 2010; 24: 419-424
19. Office for National Statistics 2011 Census; KS201EW Ethnic group: local authorities in England and Wales. Accessed 2nd May 2016 at; www.ons.gov.uk/ons/guide-method/census/2011
20. New York: State and County QuickFacts. Accessed 10th May 2016 at; www.quickfacts.census.gov/qfd/states/36000.html
21. Gondos A, Dohler B, Brenner H, Opelz G. Kidney Graft Survival in Europe and the United States: Strikingly Different Long-Term Outcomes. Transplantation 2013; 95: 267-274
22. Young CJ, Gaston RS. Understanding the Influence of Ethnicity on Renal Allograft Survival. Am J Transplant 2005; 5: 2603-2604
23. Goldfarb-Rumyantzev AS, Koford JK, Baird BC, Chelamcharla M, Habib AN, Wang BJ, et al. Role of socioeconomic status in kidney transplant outcome. Clin J Am Soc Nephrol 2006; 1: 313–322
24. Petersen E, Baird BC, Barenbaum LL, Leviatov A, Koford JK, Shihab F, et al. The impact of employment status on recipient and renal allograft survival. Clin Transplant 2008; 22: 428–438
25. Krieger N. Theories for social epidemiology in the 21st Century: an ecosocial perspective. Int J Epidemiol 2001; 30: 668-877
26. Kucirka LM, Grams ME, Lessler J, et al. Association of Race and Age With Survival Among Patients Undergoing Dialysis. JAMA 2011; 306: 620-626
27. Smith SR, Butterly DW. Declining influence of race on the outcome of living-donor renal transplantation. Am J Transplant 2002; 2: 282–286

For peer review only

For peer review only - http://bmjopen.bmj.com/site/about/guidelines.xhtml

Table 1. Baseline demographics of Black kidney transplant recipients in England versus New York State between 2003 and 2013[1]

Total		England (n=1215)	NYS (n=2660)	P-value
**Age**	Mean ± standard deviation	46.3 ± 12.4	50.1 ± 13.5	<0.001*
**Total Length of Stay**	Median ± interquartile range	10 ± (IQR 8-16)	6 ± (IQR 5-9)	<0.001**
**Post transplant hospital stay**	Median ± interquartile range	8 ± (IQR 6-13)	6 ± (IQR 4-8)	<0.001**
**Sex**	Male	743 (61.2%)	1511 (56.8%)	0.011***
	Female	472 (38.8%)	1149 (43.2%)	
**Type of Donor**	Living	343 (28.2%)	548 (20.6%)	<0.001***
	Deceased	856 (70.5%)	1555 (58.5%)	
	Unknown	16 (1.3%)	557 (20.9%)	
**Medical co-morbidities at time of transplantation**	Diabetes	194 (16.0%)	741 (27.9%)	<0.001***
	Acute MI	25 (2.1%)	99 (3.7%)	
	Cerebrovascular disease	14 (1.2%)	46 (1.7%)	
	Congestive heart failure	22 (1.8%)	220 (8.3%)	
	Liver disease	6 (0.5%)	16 (0.6%)	
	Peptic ulcer	8 (0.7%)	31 (1.2%)	
	Peripheral vascular disease	13 (1.1%)	83 (3.1%)	

[1] * t-test, ** Mann-Whitney, *** chi-squared, NYS= New York State

For peer review only - http://bmjopen.bmj.com/site/about/guidelines.xhtml

Table 2. Comparing 1-year and follow up outcomes after kidney transplantation for Black patients in England versus New York State between 2003 and 2014[2]

Variable	England	NYS	p-value
1-year follow up			
**Emergency Readmission**	782 (64.36%)	892 (33.53%)	<0.001
**Cardiac Event**	16 (1.32%)	79 (2.97%)	0.002
**Stroke**	12 (0.99%)	23 (0.86%)	<0.001
**Cancer**	17 (1.40%)	26 (0.98%)	0.245
**Fracture**	5 (0.41%)	18 (0.68%)	0.319
**Transplant Rejection/Failure**	226 (18.6%)	702 (26.4%)	<0.001
Any time Post-Transplant			
**Number of Emergency Readmissions (mean)**	4.33	3.99	0.0697
**Cardiac Event**	72 (5.93%)	336 (12.63%)	<0.001
**Stroke**	47 (3.87%)	125 (4.70%)	0.224
**Cancer**	71 (5.84%)	171 (6.43%)	0.485
**Fracture**	36 (2.96%)	84 (3.16%)	0.745
**Transplant Rejection/Failure**	367 (30.2%)	1590 (59.8%)	<0.001
Mortality			
**In Hospital Death**	9 (0.74%)	36 (1.35%)	0.099
**30 day mortality**	12 (0.99%)	61 (2.29%)	0.006
**12 month mortality**	34 (2.80%)	173 (6.50%)	<0.001

[2] Obtained using chi-squared tests

For peer review only - http://bmjopen.bmj.com/site/about/guidelines.xhtml

For peer review only

Table 3. Adjusted Logistic regression analysis of kidney transplant mortality in both England and New York State by ethnicity between 2003 and 2014[3]

	30 Day mortality			
	ENGLAND		NYS	
Ethnicity	Odds Ratio	p-value*	Odds Ratio	p-value*
Black	1 (baseline group)		1 (baseline group)	
Other	1.48 (0.76,2.88)	0.246	0.79 (0.55,1.15)	0.214
White	0.81 (0.44,1.49)	0.491	0.62 (0.44,0.86)	0.005

	1 Year Mortality			
	ENGLAND		NYS	
Ethnicity	Odds Ratio	p-value*	Odds Ratio	p-value*
Black	1 (baseline group)		1 (baseline group)	
Other	1.27 (0.85,1.90)	0.234	0.79 (0.63, 0.99)	0.064
White	0.94 (0.65, 1.34)	0.728	0.66 (0.55,0.81)	<0.001

[3] Adjusted for age, sex, admission method and number of emergency readmissions.

For peer review only - http://bmjopen.bmj.com/site/about/guidelines.xhtml

Table 4. Adjusted Cox regression analysis for transplant rejection/failure among Black kidney transplant patients in England and NYS between 2003 and 2014.

		Hazard Ratio (95% CI)	p-value
Age		1.00 (0.99-1.00)	0.171
Sex	Male	1 (baseline group)	
	Female	1.00 (0.91,1.09)	0.928
Type of Donor	Alive	1 (baseline group)	
	Dead	1.37 (1.22,1.54)	<0.001
	Unknown	1.08 (0.92,1.27)	0.339
Diabetes		1.09 (0.98,1.20)	0.114
Acute MI		0.99 (0.84,1.17)	0.935
CVF		1.04 (0.87,1.24)	0.697
PVD		1.16 (0.98,1.37)	0.086
CHF		1.16 (1.04,1.30)	0.008
Year	Pre 2007	1 (baseline group)	
	Post 2007	1.30 (1.16,1.45)	<0.001
Country	England	1 (baseline group)	
	NYS	2.15 (1.91,2.43)	<0.001

For peer review only

For peer review only - http://bmjopen.bmj.com/site/about/guidelines.xhtml

Figure legends

Figure 1. Unadjusted Kaplan Meier curve of kidney transplant rejection/failure for Black kidney transplant patients in England versus New York State between 2003 and 2014 (log-rank test p<0.001).

For peer review only

For peer review only - http://bmjopen.bmj.com/site/about/guidelines.xhtml

Figure 1. Cox regression plot of kidney transplant rejection/failure for Black kidney transplant patients in England versus New York State

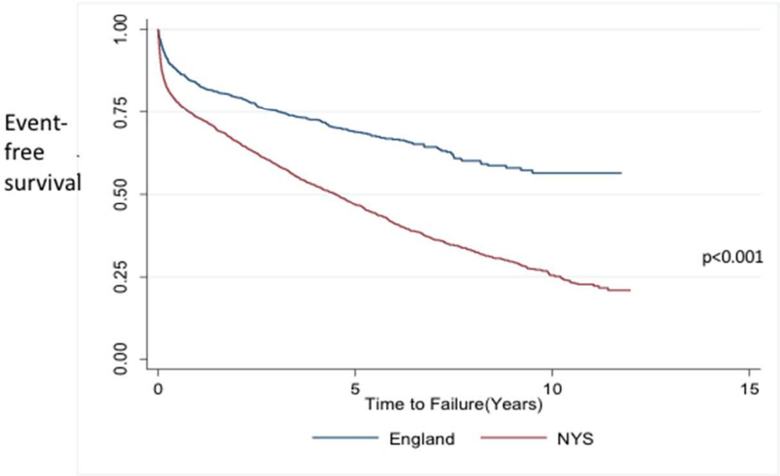

338x190mm (54 x 54 DPI)

review only

Supplementary Tables showing sensitivity analyses

		30 Day Mortality					
		England			NYS		
		Pre-Sensitivity Analysis	Black and Unknown combined	White and unknown combined	Pre-Sensitivity Analysis	Black and Unknown combined	White and unknown combined
Age Category	<50	1 (baseline group)	1 (baseline group)	1 (baseline group)	1 (baseline group)	1 (baseline)	1 (baseline)
	50+	3.76 (2.64,5.37)	3.76 (2.64,5.36)	3.76 (2.64,5.37)	1.21 (0.91,1.61)	1.21 (0.91,1.61)	1.21 (0.91,1.61)
Sex	Male	1 (baseline group)	1 (baseline group)	1 (baseline group)	1 (baseline group)	1 (baseline)	1 (baseline)
	Female	1.12 (0.82,1.54)	1.12 (0.82,1.54)	1.13 (0.82,1.55)	0.88 (0.66,1.17)	0.88 (0.66,1.17)	0.88 (0.66,1.17)
Admission Method	Elective	1 (baseline group)	1 (baseline group)	1 (baseline group)	1 (baseline group)	1 (baseline)	1 (baseline)
	Emergency	2.47 (1.75,3.50)	2.47 (1.74,3.49)	2.47 (1.75,3.49)	2.00 (1.49,2.68)	2.00 (1.49,2.68)	2.00 (1.49,2.68)
	Other	0.51 (0.07,3.79)	0.51 (0.07,3.73)	0.52 (0.07,3.86)	2.52 (0.59,10.71)	2.53 (0.60,10.74)	2.53 (0.60,10.75)
Number of Emergency Readmissions		0.20 (0.15,0.27)	0.20 (0.15,0.27)	0.20 (0.15,0.27)	0.77 (0.72,0.83)	0.77 (0.72,0.83)	0.77 (0.72,0.83)
Ethnic Group	Black	1 (baseline group)	1 (baseline group)	1 (baseline group)	1 (baseline group)	1 (baseline)	1 (baseline)
	Other	1.48 (0.76,2.88)	1.60 (0.97,2.63)	1.43 (0.73,2.82)	0.79 (0.55,1.15)	0.79 (0.54,1.14)	0.79 (0.54,1.15)
	White	0.81 (0.44,1.49)	0.79 (0.52,1.19)	0.73 (0.39,1.34)	0.62 (0.44,0.86)	0.61 (0.44,0.85)	0.62 (0.45,0.87)

		12 Month Mortality					
		England			NYS		
		Pre-Sensitivity Analysis	Black and Unknown combined	White and unknown combined	Pre-Sensitivity Analysis	Black and Unknown combined	White and unknown combined
Age Category	<50	1 (baseline group)	1 (baseline)	1 (baseline)	1 (baseline group)	1 (baseline)	1 (baseline)
	50+	3.97 (3.24,4.86)	3.94 (3.22,4.83)	3.97 (3.24,4.86)	1.45 (1.21,1.73)	1.21 (0.91,1.61)	1.21 (0.91,1.61)
Sex	Male	1 (baseline group)	1 (baseline)	1 (baseline)	1 (baseline group)	1 (baseline)	1 (baseline)
	Female	1.07 (0.89,1.27)	1.06 (0.89,1.27)	1.06 (0.89,1.27)	1.06 (0.89,1.26)	0.88 (0.66,1.17)	0.88 (0.66,1.17)
Admission Method	Elective	1 (baseline group)	1 (baseline)	1 (baseline)	1 (baseline group)	1 (baseline)	1 (baseline)
	Emergency	1.84 (1.53,2.22)	1.85 (1.54,2.23)	1.84 (1.52,2.22)	1.79 (1.50,2.13)	2.00 (1.49,2.68)	2.00 (1.49,2.68)
	Other	1.17 (0.59,2.33)	1.19 (0.60,2.37)	1.18 (0.59,2.33)	1.76 (0.62,4.98)	2.53 (0.60,10.74)	2.53 (0.60,10.75)
Number of Emergency Readmissions		0.84 (0.82,0.87)	0.84 (0.81,0.87)	0.84 (0.81,0.87)	0.89 (0.86,0.92)	0.77 (0.72,0.83)	0.77 (0.72,0.83)
Ethnic Group	Black	1 (baseline group)	1 (baseline)	1 (baseline)	1 (baseline group)	1 (baseline)	1 (baseline)
	Other	1.24 (0.85,1.90)	1.60 (1.17,2.18)	1.27 (0.84,1.91)	0.79 (0.63,0.99)	0.79 (0.54,1.14)	0.79 (0.54,1.15)
	White	0.94 (0.65,1.34)	1.19 (0.92,1.54)	0.91 (0.63,1.32)	0.66 (0.55,0.81)	0.61 (0.44,0.85)	0.62 (0.45,0.87)

Living and Unknown Combined

		Hazard Ratio (95% CI)	p-value
Age		1.00 (0.99,1.00)	0.181
Sex	Male	1 (baseline group)	
	Female	0.99 (0.91,1.09)	0.897
Type of Donor	Alive/unknown	1 (baseline group)	
	Dead	1.33 (1.20,1.46)	<0.001
Diabetes		1.08 (0.98,1.20)	0.12
Acute MI		0.99 (0.85,1.17)	0.945
CVF		1.03 (0.87,1.23)	0.716
PVD		1.16 (0.98,1.38)	0.086
CHF		1.16 (1.04,1.30)	0.008
Year	Pre 2007	1 (baseline group)	
	Post 2007	1.27 (1.15,1.41)	<0.001
Country	England	1 (baseline group)	
	NYS	2.17 (1.93,2.45)	<0.001

Deceased and Unknown Combined

		Hazard Ratio (95% CI)	p-value
Age		1.00 (0.99,1.00)	0.24
Sex	Male	1 (baseline group)	
	Female	1.00 (0.91,1.09)	0.96
Type of Donor	Alive	1 (baseline group)	
	Dead/Unknown	1.31 (1.16,1.47)	<0.001
Diabetes		1.09 (0.98,1.20)	0.103
Acute MI		0.99 (0.85,1.17)	0.945
CVF		1.04 (0.87,1.24)	0.655
PVD		1.17 (0.98,1.38)	0.077
CHF		1.18 (1.06,1.32)	0.004
Year	Pre 2007	1 (baseline group)	
	Post 2007	1.41 (1.27,1.56)	<0.001
Country	England	1 (baseline group)	
	NYS	2.06 (1.83,2.32)	<0.001

Alive donors only

		Hazard Ratio (95% CI)	p-value
Age		0.98 (0.97,0.99)	<0.001
Sex	Male	1 (baseline group)	
	Female	1.13 (0.92,1.39)	0.248
Diabetes		1.25 (0.98,1.58)	0.071
Acute MI		1.12 (0.74,1.70)	0.596
CVF		0.64 (0.41,1.02)	0.058
PVD		1.56 (1.05,2.31)	0.028
CHF		1.29 (0.99,1.69)	0.058
Year	Pre 2007	1 (baseline group)	
	Post 2007	1.34 (1.05,1.72)	0.02
Country	England	1 (baseline group)	
	NYS	1.68 (1.32,2.13)	<0.001

Deceased donors only

		Hazard Ratio (95% CI)	p-value
Age		1.00 (1.00,1.00)	0.915
Sex	Male	1 (baseline group)	
	Female	1.00 (0.90,1.13)	0.934
Diabetes		1.10 (0.97,1.25)	0.144
Acute MI		0.96 (0.79,1.16)	0.645
CVF		1.15 (0.94,1.41)	0.178
PVD		1.07 (0.87,1.31)	0.515
CHF		1.13 (0.99,1.30)	0.075
Year	Pre 2007	1 (baseline group)	
	Post 2007	1.25 (1.09,1.43)	0.001
Country	England	1 (baseline group)	
	NYS	2.25 (1.95,2.59)	<0.001

For peer review only - http://bmjopen.bmj.com/site/about/guidelines.xhtml

BMJ Open

Do outcomes after kidney transplantation differ for Black patients in England versus New York State? A comparative, population-cohort analysis

Journal:	*BMJ Open*
Manuscript ID	bmjopen-2016-014069.R2
Article Type:	Research
Date Submitted by the Author:	14-Feb-2017
Complete List of Authors:	Tahir, Sanna<br>Gillott, Holly<br>Jackson-Spence, Francesca<br>Nath, Jay<br>Mytton, Jemma<br>Evison, Felicity; University Hospitals Birmingham NHS Foundation Trust, Department of Informatics<br>Sharif, Adnan
**Primary Subject Heading**:	Renal medicine
Secondary Subject Heading:	Health services research, Renal medicine, Epidemiology
Keywords:	TRANSPLANT MEDICINE, EPIDEMIOLOGY, International health services < HEALTH SERVICES ADMINISTRATION & MANAGEMENT, Quality in health care < HEALTH SERVICES ADMINISTRATION & MANAGEMENT

SCHOLARONE™
Manuscripts

Title. Do outcomes after kidney transplantation differ for Black patients in England versus New York State? A comparative, population-cohort analysis

Author. Sanna Tahir[1], Holly Gillott[1], Francesca Jackson Spence[1], Jay Nath[1,2], Jemma Mytton[3], Felicity Evison[3], Adnan Sharif[2,4]

Departments and Institutions.

[1]University of Birmingham, Birmingham, UK

[2]Department of Nephrology and Transplantation, Queen Elizabeth Hospital, Edgbaston, Birmingham, UK

[3]Department of Health Informatics, Queen Elizabeth Hospital, Edgbaston, Birmingham, UK

[4]School of Immunology and Inflammation, University of Birmingham, Birmingham, UK

Corresponding author contact information.

Dr. Adnan Sharif

Department of Nephrology and Transplantation

Queen Elizabeth Hospital, Edgbaston, Birmingham, B15 2WB, United Kingdom

Phone: (0121 371 5861), Fax: (0121 472 4942), Email: adnan.sharif@uhb.nhs.uk

Running title: Comparing Black post-transplant outcomes

Word count: Abstract (300), Main (3995)

For peer review only

Abbreviations

ESKD – End-stage kidney disease,

NYS – New York State

SPARCS - Statewide Planning and Research Cooperative System

HES – Hospital Episode Statistics

OPCS-4 – Classification of Interventions and Procedures version 4

ICD - International Classification of Diseases

For peer review only

For peer review only - http://bmjopen.bmj.com/site/about/guidelines.xhtml

Abstract

Objectives. Inferior outcomes for Black kidney transplant recipients in the United States may not be generalizable elsewhere. In this population-cohort analysis, we compared outcomes for Black kidney transplant patients in England versus New York State.

Design. Retrospective, comparative, population cohort study utilizing administrative data registries.

Settings and Participants. English data was derived from Hospital Episode Statistics, while New York State data was derived from Statewide Planning and Research Cooperative System. All adults receiving their first kidney-alone allograft between 2003 and 2013 were eligible for inclusion.

Measures. The primary outcome measure was mortality post kidney transplantation (including in-hospital death, 30-day and 1-year mortality). Secondary outcome measures included post-operative admission length of stay, risk of re-hospitalization, development of cardiac events, stroke, cancer or fracture and finally transplant rejection/failure. Cox proportional hazards regression was used to investigate relationship between ethnicity, country and outcome.

Results. Black patients comprised 6.5% of the English cohort (n=1215/18,493) and 23.0% of the New York State cohort (n=2660/11,602). Compared to New York State, Black kidney transplant recipients in England were more likely younger, male, living-donor kidney recipients and had dissimilar medical co-morbidities. Inpatient mortality was not statistically different but death within 30-days, 1-year or kidney transplant rejection/failure was lower among black patients in England versus Black patients in New York State. In adjusted regression analysis, with Black ethnicity the reference group, White patients had reduced risk for 30-day mortality (odds ratio 0.62 [95% CI 0.44-0.86]) and 1-year mortality (odds ratio

For peer review only - http://bmjopen.bmj.com/site/about/guidelines.xhtml

0.79 [95% CI 0.63-0.99]) in New York State but no difference was observed in England. Compared to England, Black kidney transplant patients in New York State had increased hazard ratio for kidney transplant rejection rejection/failure by median follow up (hazard ratio 2.15, 95% CI 1.91-2.43).

Conclusions. Outcomes after kidney transplantation for Black patients may not be translatable between countries.

For peer review only

Article summary

Strengths and limitations of this study

- Large population-cohort study of two
- Data linkage to mortality data for accuracy
- Use of administration rather than registry data
- Kidney allograft survival data not well defined
- Numerous confounders unavailable to robustly probe this question

For peer review only - http://bmjopen.bmj.com/site/about/guidelines.xhtml

Introduction

Black patients are significantly more likely to develop end-stage kidney disease (ESKD), which is likely secondary to increased risk of chronic kidney disease progressing to kidney failure (1). Adjusted incident rates for Black patients (or African Americans) in the United States Renal Data System (USRDS) in 2014 (latest available data) was the highest for any ethnicity group, with a EDKD incidence rate ratio versus White patients of 3.1 (2). Even in countries with a lower incidence of ESKD than the United States like the United Kingdom, there is a disproportionate number of Black patients with ESKD undergoing renal replacement therapy compared to the general population (3). Therefore, strategies to optimise care for Black patients with ESKD remains of critical importance.

While kidney transplantation is recognised as the preferred modality of renal replacement therapy for patients with ESKD, disparate post-transplant outcomes are reported for Black recipients of a kidney allograft. For example, Black kidney transplant patients in the United States have previously been shown to have worse kidney allograft survival compared to White recipients (4), although recent analyses suggest reduced disparity (but still not equality) in kidney allograft outcomes between Black and White patients in the contemporary era in the United States (5). However, this is not generalizable across North America as Canadian Black transplant patients appear to not only have similar kidney allograft survival but improved mortality compared to Canadian White kidney transplant patients (6). Mortality differences after kidney transplantation between the United States and Canada are well recognised, with magnification of risk for death observed in Black Americans versus Canadians (7). In a European setting, Pallet and colleagues have shown no difference in outcomes for Black versus White patients after kidney transplantation in

For peer review only - http://bmjopen.bmj.com/site/about/guidelines.xhtml

For peer review only

France (8) and no mortality difference is observed between Black and White patients in the United Kingdom (9, 10). Therefore, inferior mortality observed for Black kidney transplant patients widely attributed to the United States may be country-specific, thus making interpretation of such data difficult for other population cohorts like the United Kingdom.

No comparative analysis has ever been conducted to specifically compare post-transplant outcomes for Black kidney transplant patients between countries across distinct geographical regions like the United Kingdom and the United States. This is of interest as healthcare systems differ significantly between the two countries, with the possibility that Black people in the United Kingdom may not share the same post-transplant outcomes observed in the United States. Therefore, it is unclear if the counselling of Black kidney transplant candidates in one country can be adequately made utilizing data translated from Black patients in another country. The aim of this analysis was to undertake a comparative population-based cohort analysis exploring outcomes after kidney transplantation for Black patients in England versus New York State.

Materials and Methods

Study population

We obtained data from every patients' first kidney-alone transplant procedure performed in England and New York State between 2003 and 2013, collecting patient demographics that included age, gender, donor type (living or deceased), transplant year, medical co-morbidities (based upon ICD-10 codes) and ethnicity. English data was obtained from Hospital Episode Statistics (11), an administrative data warehouse containing admissions to all National Health Service hospitals in England. It contains detailed records relating to

For peer review only

For peer review only - http://bmjopen.bmj.com/site/about/guidelines.xhtml

individual patient treatments; with data extraction facilitated utilizing codes on procedural classifications (Office of Population Censuses and Surveys Classification of Interventions and Procedures, 4th revision [OPCS-4]) (12) and medical classifications (World Health Organization International Classification of Disease, 10th revision [ICD-10]) (13). The comparative analysis with the United States was performed with contemporaneous New York State data and extracted from the Statewide Planning and Research Cooperative System (SPARCS), a comprehensive all payer data reporting system collecting patient level data across New York State (14). The database collects information including patient demographics, diagnoses, procedures, and charges for every inpatient hospital admission, ambulatory surgical procedure, and emergency department admission. Individuals are assigned a unique, encrypted identification code, allowing for longitudinal analyses. Estimated reporting completeness obtained from SPARCS inpatient annual reports during the study period (2000-11) ranged from 95% to 100%, with an average of more than 98%.

This study included all patients' first kidney transplant procedures (OPCS-4 codes; M01[0-5,8,9]) performed in England and New York State between the years of 2003 to 2013. With regards to outcome analysis, both HES and SPARCS datasets have the limitation of only capturing deaths occurring in a hospital setting. To obtain the complete mortality list the study cohort was cross-referenced with mortality data from the Office for National Statistics and New York State/New York City Vital Statistics respectively, which collects information on all registered deaths in the United Kingdom and New York State respectively. Death data was available from 1st January 20103 up until 31st December 2014. Combining sources via this data linkage process creates a comprehensive dataset with regards to mortality, which was the endpoint of interest in this analysis. In addition, we extracted data for kidney

For peer review only

transplant rejection/failure (ICD-10; T861 or ICD-9; 99681), which is derived from the administrative data and substituted for lack of data linkage to transplant registry records. Formal ethical approval was not required due to the pseudonymised nature of the data retrieved; data was linked by NHS Informatics utilizing a special HES ID code and avoided any patient identifiable information. Thus study was registered as an audit with University Hospitals Birmingham NHS Trust (audit identifier; CARMS-12578).

Data inclusion

We extracted data on all adult kidney allograft recipients' first procedure between the dates of 1st January 2003 and 31st December 2013, who underwent their kidney transplant procedure in a transplant center in either England or New York State. From our original cohorts of 21,371 and 12,373 patients in England and New York States, we excluded from analysis 2,582 and 771 patients from both data sets respectively as shown in Supplementary Figure 1. Ethnicity status was missing in <5 cases (too small to identify), although anyone classified as unknown was included under 'other' for analysis. All patients were followed up until December 2014.

Outcomes

The primary outcome measure was all-cause mortality post kidney transplantation (including in-hospital death, 30-day and 1-year mortality). In addition, we looked at various secondary outcome measures including; post-operative admission length of stay, risk of re-hospitalization, development of cardiac events (ICD10: I2[01234] , ICD9: 410,411,413,429), stroke (ICD10: I6[0123456], ICD9: 430-436), cancer (ICD10:C00-C99, ICD9: 140-199) or fracture (ICD10: S[0123456789]2, T02, T07, T10, T12, ICD9: 800-829) and finally kidney

For peer review only - http://bmjopen.bmj.com/site/about/guidelines.xhtml

transplant rejection/failure (ICD10: T861, ICD9: 99681). Re-hospitalisation was defined as whether a patient was readmitted to hospital as an emergency at any time post-discharge of their transplant admission. All other secondary outcomes were captured with use of ICD10 and ICD9 codes (as defined above), at any time post-transplant.

Statistical analysis

Differences between groups were compared using chi-square tests for categorical variables two-sample t-test for normal continuous variables or Mann-Whitney tests for all non-normal continuous variables. Multivariate logistic regression was used to measure 30 day and 12 month mortality. All patients were included in these models to see if there was a difference in mortality outcomes for the different ethnic groups, within the 2 populations. Variables included in the models were age, sex, admission method (emergency v elective), number of emergency readmissions and ethnic group. Survival analyses were performed, combining all black patients in the two countries, for the outcome of graft failure/rejection (defined using ICD10 T861 or ICD 99681 at any time post-transplant) using Cox's proportional hazards model and the generation of Kaplan Meier plots. The proportional hazard assumption was checked and satisfied by examination of the scaled Schoenfeld residual plots. Variables included in the cox model were ethnicity, age, gender, donor type (living versus deceased), year of transplant, geography (England versus New York State) and selected medical co-morbidities at the time of transplant (history of myocardial infarction, peripheral vascular disease, cerebrovascular disease, congestive cardiac failure and diabetes). A *p* value less than 0.05 was considered statistically significant in the analysis. All statistical analyses were conducted using Stata 14, StataCorp LP, Texas, USA.

For peer review only - http://bmjopen.bmj.com/site/about/guidelines.xhtml

Results

Between 2003 and 2013 there were 21,371 patients in England and 12,373 patients in New York State who had their first Kidney Transplant. After exclusions due to missing age or sex, and those who had multi-organ transplants, data was analysed for 18,493 and 11,602 patients between 2003 and 2013 from England and New York State respectively, with median follow up in England and New York State 6.3 years and 5.5 years respectively. Black patients comprised 6.5% of the English cohort (n=1215) and 23.0% of the New York State cohort (n=2660), forming the basis of all comparative analyses.

Baseline demographics

Table 1 compares demographics between Black patients in England versus New York State. Black kidney allograft recipients in England versus New York State were found to be younger (46.1 versus 50.1 respectively, p<0.001), more likely to be male (61.2% versus 56.8% respectively, p=0.011) and more likely to get living-donor kidneys (28.2% versus 20.6% respectively, p<0.001). We also observed Black recipients in England versus New York State had less medical co-morbidities listed at time of transplantation including diabetes (16.0% versus 27.9% respectively, p<0.001), previous myocardial infarcts (2.1% versus 3.7% respectively, p<0.001), strokes (1.2% versus 1.7% respectively, p<0.001) and congestive cardiac failure (1.8% versus 8.0% respectively, p <0.001), suggesting a higher level of medical co-morbidity listed at the time of kidney transplantation for Black patients in New York State versus England.

For peer review only - http://bmjopen.bmj.com/site/about/guidelines.xhtml

Post-transplant outcomes

Table 2 demonstrates the difference in mortality outcomes comparing Black kidney transplant patients in England versus New York State. While inpatient mortality was similar comparing Black patients in England versus New York State (0.7% versus 1.4% respectively, p=0.099), mortality was significantly lower among Black patients in England versus New York State within 30-days (1.0% versus 2.3% respectively, p=0.006) and 1-year (2.8% versus 6.5% respectively, p<0.001)) after kidney transplantation.

There were significant differences in the post-transplantation course for Black kidney transplant patients in England versus New York State (see Tables 1 and 2). Black patients in England versus New York State had longer post-operative inpatient stays (Median 8 versus 6 days, p <0.001) and had a greater tendency to be readmitted within the first year post-transplant (64.36% versus 33.53%, p<0.001), but overall mean number of emergency re-hospitalisation episodes' post-transplant by median follow up was similar between the two cohorts (4.3 versus 4.0 respectively, p=0.07). With regards to medical events occurring post kidney transplantation, Black patients in England versus New York State were less likely to have cardiac events (6.0% versus 12.6% respectively, p<0.001), but no difference was observed in episodes of cancer, strokes or fractures after kidney transplantation for Black patients in England versus New York State.

Regression analysis

While our analyses revealed significant mortality differences between Black patients in England versus New York State, we were keen to understand if Black ethnicity itself was a

For peer review only - http://bmjopen.bmj.com/site/about/guidelines.xhtml
For peer review only

risk factor for mortality in each population cohort. In a logistic regression model, accounting for variables associated with mortality after kidney transplantation (age, sex, admission method, number of emergency readmissions), we analysed whether Black ethnicity was an independent predictor for death in England or New York State. With Black ethnicity utilised as the reference group, Table 3 displays the output from the logistic regression analysis and shows White kidney allograft recipients had reduced risk for 30-day mortality (odds ratio 0.62 [95% confidence interval 0.44-0.86]) and 1-year mortality (odds ratio 0.66 [95% confidence interval 0.55-0.81]) in New York State but no difference was observed in England (30-day mortality OR 0.81 (0.44-1.49), 1-year mortality OR 0.94 (0.65-1.34).

A significant difference was observed in unadjusted Kaplan-Meier plots of kidney transplant rejection/failure for Black kidney transplant patients in England versus New York States (see Figure 1). After adjustment for baseline variables, Cox regression analysis confirmed Black kidney transplant patients in New York State versus England had over the double the hazard ratio for kidney transplant rejection/failure during follow up (hazard ratio 2.15, 95% CI 1.91-2.43) (see Table 4), which was identical to a similar analysis focusing only on White patients (see Supplementary Table 1).

Discussion

Our comparative analysis of two large population-base cohort datasets demonstrates Black kidney transplant patients have different baseline characteristics and post-transplant outcomes in England compared to New York State. Most importantly, Black patients in England have lower mortality than their New York State counterparts, where Black ethnicity

For peer review only - http://bmjopen.bmj.com/site/about/guidelines.xhtml

in the latter cohort was found to be independently associated with increased 30-day and 1-year mortality. Our analysis, to our knowledge, is the first comparative study comparing Black kidney allograft outcomes in contemporaneous population cohorts. Our study highlights significant differences between Black kidney transplant patients in England versus New York State and suggests caution in translating outcomes for Black transplant patients between different countries.

The strengths and weaknesses of this study must be appreciated for the correct interpretation of our results. There are likely to be numerous confounders that have an impact on Black mortality post kidney transplantation that we were unable to factor in (e.g. smoking, lifestyle, socio-cultural factors, dialysis vintage etc.). It should be acknowledged that New York State data may not be completely representative of other States in the United States and we are crudely inferring that data from New York State is broadly representative of the United States. Missing data (and misclassification bias) also has an implication on the analyses performed and this limitation is inherent with all epidemiological studies of this type. Unfortunately, while the majority of data obtainable was matched between the two cohorts, we were unable to extract any information regarding socioeconomic deprivation status or cause of death from the SPARCS dataset. Our utilization of the composite for kidney transplant rejection/failure from administration records is an inferior surrogate compared to registry-derived data regarding kidney allograft rejection and graft failure. The ability to extract more data, and to link datasets to create more comprehensive databases, allows the minimization of confounding and provides a more robust platform from which to conduct meaningful epidemiological analysis. Further work in this area is clearly warranted and should be undertaken using more robust renal

For peer review only - http://bmjopen.bmj.com/site/about/guidelines.xhtml

data including registry data from both the United States (e.g. US Renal Data System, Scientific registry of Transplant Recipients) and the United Kingdom (e.g. UK Transplant Registry, Renal Registry). Finally, mortality data is readily available and was the sole analytical endpoint of this analysis. However, absence of recorded death may not necessarily translate to assumption that a recipient remains alive (e.g. lost to follow up due to emigration).

Current literature suggests Black kidney transplant patients in the United States have inferior kidney allograft survival compared to White recipients (4) and risk for death is magnified among Black American versus Canadian kidney transplant patients (7). Reassuringly, recently published work demonstrates significant improvement in post kidney transplant outcomes in the United States over the last two decades, more so for Black versus White patients, leading to reduced disparity (5). This may be related to advances in immunosuppression and post-transplant management, possibly benefiting Black kidney transplant patients more significantly who perhaps were more disproportionately overburdened by immunological barriers in the past. While some disparity remained in their analysis in relation to significantly raised adjusted hazard ratios for Black patients and risk for graft loss in the contemporary setting, the vastly improved outcomes were encouraging for transplant clinicians to actively promote kidney transplantation among Black patients. Our analysis suggested worse kidney allograft outcomes since 2007 for all Black kidney transplant recipients, which may be due to more high-risk kidney transplantation of medically complex or immunological difficult patients, but this requires further analysis with more transplant-specific data as it may simply reflect better administrative coding of kidney rejection/failure since 2007.

For peer review only - http://bmjopen.bmj.com/site/about/guidelines.xhtml

By contrast, no mortality difference between Black and White patients has been observed in the few limited publications within European transplant centres (8-10). Our data therefore supports the inference from existing literature about disparate outcomes for Black patients in the United States versus elsewhere and raises two important questions; 1) why outcomes for Black transplant patients in the United States remain inferior to White patients, and; 2) why Black transplant patients in the United States have inferior outcomes in comparison to Black patients outside of the United States. It could be argued that the Black population in England differs from the United States, but this is an insufficient explanation as Black people in Canada and the United States share geographical origins but still have dissimilar mortality outcomes (7). It is important to distinguish the significant differences in baseline demographics between our two Black cohorts. For example, medical co-morbidities such as history of diabetes and atherosclerotic diseases were less common among Black transplant recipients in England compared to New York State, suggesting a higher level of cardiovascular burden at baseline for the latter. This could explain the significantly increased risk for cardiac events within the first year after kidney transplantation for Black kidney transplant patients in New York State versus England. Due to the absence of cause of death from the SPARCS data, we were unable to ascertain the nature of deaths among Black patients in New York State but an increase in cardiac-deaths would be a reasonable assumption. However, baseline demographics alone cannot account for the significant difference in mortality.

Perhaps the most fundamental difference between countries is the infrastructure delivering and providing healthcare. Black kidney transplant patients in England have universal health

For peer review only - http://bmjopen.bmj.com/site/about/guidelines.xhtml

coverage as part of the taxpayer-funded National Health Service. This ensures complete financial access not just to immunosuppressive medications but also other aspects of care including allied medications (e.g. anti-hypertensive agents, glucose-lowering agents, lipid-lowering drugs, etc.) and long-term medical follow up to support post-transplant care. Compared to the United States, countries such as England, Canada and France that demonstrate similar mortality outcomes after transplantation for Black versus White patients share elements of universal health coverage (6, 8-10) and this is an obvious factor to explain disparate mortality outcomes. This is also supported by our analysis also showing improved graft survival for White patients in England versus New York States, suggesting our analysis reflects better outcomes overall in England rather than any ethnicity effect. However, universal health coverage alone is unlikely to be the sole explanation for differing outcomes. Prospective clinical trials in the United States, which may attenuate the risk of any lack of access, still demonstrate poorer outcomes for Black patients in sub-group analyses (15), suggesting health coverage alone does not fully explain poor outcomes. This is supported by evidence within the United States shown by Chakkera and colleagues in their exploration of racial disparities using kidney transplant cohorts, which included patients receiving health care within versus outside the Department of Veterans Affairs (VA) (16). The VA is the largest integrated managed healthcare system in the United States and provides comprehensive medical care to eligible veterans including coverage of prescription drugs. Perhaps surprisingly, the association of Black ethnicity with poorer outcomes was consistent in non-VA versus VA users, with the latter associated with increased risk of graft loss (adjusted graft failure 1.31, 95% CI 1.10-1.57) and death (adjusted mortality 1.10, 95% CI 0.90-1.34). While noting adjusted mortality for VA users was not significantly raised in the analysis (likely due to a smaller cohort of 1,646 VA users versus 77,715 non-VA users), the

For peer review only

For peer review only - http://bmjopen.bmj.com/site/about/guidelines.xhtml

adjusted risks for graft failure and death were almost identical between VA and non-VA users (1.31 and 1.31 respectively for graft failure; 1.10 and 1.11 respectively for mortality).

This raises the question as to what underlies poor outcomes among Black kidney transplant patients in the United States, while acknowledging disparate outcomes appear to be narrowing between Black and White patients post kidney transplantation (5). Factors such as non-adherence, which could be a surrogate for non-affordability of drugs in the United States, is a phenomenon that is common in transplantation cohorts everywhere (including countries with universal health coverage) (17). There could be selection bias from poor access to transplantation for Black people, which has been acknowledged in transplant centers across the world, and has been comprehensively reviewed elsewhere (18). However, from the most up-to-date census reports, individuals of Black ethnicity make up 3.5% of the English population (19) and 17.6% of the New York State population (20) and Black patients formed 6.5% and 23.0% of our transplant cohorts respectively. While this suggests Black patients are over-represented as kidney allograft recipients in both England and New York State respectively, it must be remembered that ESKD is more prevalent in Black communities and therefore they are more likely to need renal replacement therapy. In should be noted that disparate post-transplant outcomes between Europe and the United States are not unique to kidney allograft recipients of Black ethnicity alone. Gondos and colleagues, analysing data from the United Network for Organ Sharing (UNOS) and the Collaborative Transplant Study (CTS), compared kidney transplant outcomes in the United States versus Europe respectively (21). Adjusted hazard ratios for graft loss were higher in the United States compared to Europe and identifying the contributing factors to this disparity should be considered a high priority to improve the delivery of post-transplant

For peer review only - http://bmjopen.bmj.com/site/about/guidelines.xhtml

care. Socioeconomic status, education and poverty are all likely to be important and Black transplant patients in the United States may an inherent risk for higher mortality or have weaker social support networks or negative health behavior that drives risk for adverse outcomes (such as smoking, alcohol consumption, sedentary lifestyle and poor diet) (22-24). It is clear our understanding of the social epidemiological impact on health inequalities is limited (25), but deepening our understanding of these issues may further reduce the disparity in mortality outcomes seen in the United States for Black kidney allograft recipients.

Our analysis should be interpreted in the context of the well recognized observation of reduced risk for death for Black versus White ESKD patients after commencement of dialysis and this phenomenon has been noted in both the United States (2) and the United Kingdom (3). However, inferring that *all* Black patients on dialysis have reduced risk for death on dialysis may be incorrect when interpreting large population cohorts in the context of competing factors. For example, Kucirka and colleagues conducted an observational cohort study of 1,330,007 incident end-stage renal disease patients in the United States Renal Data System (USRDS) and showed Black patients have lower mortality on dialysis than White patients (adjusted hazard ratio 0.84; 95% confidence interval 0.83-0.84; $p<0.001$) (26). However, after stratification by age and treating kidney transplantation as a competing risk, Black patients below the age of 50 had significantly *higher* mortality than their White counterparts on dialysis. Teasing out the differences that help explain our disparate mortality outcomes between Black patients in England versus New York State will likely prove more challenging than just a simple explanation. Caution must also be exercised in the interpretation of epidemiological data to ensure false inferences are not made from

For peer review only - http://bmjopen.bmj.com/site/about/guidelines.xhtml

For peer review only

statistical analyses. For example, Yeates and colleagues found Black kidney transplant patients in Canada had significantly lower post-transplantation mortality compared with White patients (hazard ratio 0.49; 95% confidence interval 0.28-0.88; p=0.02) (6). This finding is surprising, and the only documented example of this observation, which requires corroboration before any legitimate interpretation can be made. However, there are recognized steps that can be taken to reduce the risk of post-transplant mortality for Black patients including greater use of living-kidney donors, which has been shown to improve outcomes and reduces racial disparity of post-transplant outcomes (27).

To conclude, this is the first comparative study examining mortality for Black kidney transplant patients between two different countries and found Black kidney transplant patients in New York State have a greater hazard for death and kidney transplant failure/rejection compared to their Black counterparts in England. Our results suggest further work is essential to investigate poor outcomes for Black patients after kidney transplantation in the United States, to aid our inadequate understanding of their social, environmental and/or biological pressures. While macro-level factors like universal health coverage may be an important factor contributing to post-transplantation mortality and allograft loss for Black patients, our study suggests additional micro-level factors are likely to be concomitantly important and are in need of deeper evaluation.

Funding

We are grateful for the funding in support of the BMedSci intercalated degrees for ST and FJS (from Kidney Research UK) and HG (from Arthur Thomson Trust).

For peer review only - http://bmjopen.bmj.com/site/about/guidelines.xhtml

Competing interests statement

We have read and understood BMJ policy on declaration of interests and declare that we have no competing interests.

Contributorship statement

Designed study[ST,JN,AS], Data extraction[ST,HG,FJS,JM,FE], Data analysis[ST,JM,FE], Data interpretation[ST,JN,AS], Wrote original draft[ST,AS], Reviewed manuscript[ST,HG,FJS,JN,JM,FE,AS]

Data sharing statement

No additional data available

References

1. Hsu CY, Lin F, Vittinghoff E, Shlipak MG. Racial Differences in the Progression from Chronic Renal Insufficiency to End-Stage Renal Disease in the United States. J Am Soc Nephrol 2003; 14: 2902-2907
2. United States Renal Data System. Chapter 1; Incidence, prevalence, patient characteristics & mortality. Accessed 12th February 2016; www.usrds.org/2016/view/v2_01.aspx
3. UK Renal Registry, 2014 – The Seventeenth Report. Accessed 5th February 2016 at; www.renalreg.org/reports/2014-seventeenth-annual-report/
4. Isaacs RB, Nock SL, Spencer CE, Connors AF Jr, Wang XQ, Sawyer R, et al. Racial disparities in renal transplant outcomes. Am J Kidney Dis 1999; 34: 706–712
5.]Purnell TS, Massie AB, Boulware LE, et al. Reduced Racial Disparity in Kidney Transplant Outcomes in the United States from 1990 to 2012. J Am Soc Nephrol 2016; published ahead of print
6. Yeates K, Wiebe N, Gill J, et al. Similar Outcomes among Black and White Renal Allograft Recipients. J Am Soc Nephrol 2009; 20: 172-179
7. Kim SJ, Schaubel DE, Fenton SSA, Leichtman AB, Port FK. Mortality After Kidney Transplantation: A Comparison Between the United States and Canada. Am J Transplant 2006; 6: 109-114
8. Pallet N, Thervet E, Alberti C, et al. Kidney transplant in black recipients: are African Europeans different from African Americans? Am J Transplant 2005; 5: 2682-7

For peer review only - http://bmjopen.bmj.com/site/about/guidelines.xhtml

For peer review only

9. Rudge C, Johnson RJ, Fuggle SV, et al. Renal transplantation in the United Kingdom for patients from ethnic minorities. Transplantation 2007; 83: 1169-73
10. Medcalf JF, Andrews PA, Bankart J, et al. Poorer graft survival in ethnic minorities: results from a multi-centre UK study of kidney transplant outcomes. Clin Nephrol 2011; 75: 294-301
11. Health & Social Care Information Centre: Hospital Episode Statistics online. Accessed on 6th May 2016 at; www.hscic.gov.uk/hes
12. OPCS-4 Classification – NHS Connecting for Health. Accessed on 5th February 2016; www.connectingforhealth.nhs.uk
13. World Health Organization: International Classification of Diseases (ICD). Accessed accessed 5th May 2016 at; www.who.int/classifications/icd/en
14. Statewide Planning and Research Cooperative System (SOARCS). Accessed 2nd May 2016 at; www.health.ny.gov/statistics/spacrs/
15. Woodle ES, First MR, Pirsch J, et al. A Prospective, Randomized, Double-Blind, Placebo-Controlled Multicenter Trial Comparing Early (7 Day) Corticosteroid Cessation Versus Long-Term, Low-Dose Corticosteroid Therapy. Ann Surg 2008; 248: 564-77
16. Chakkera HA, O'Hare AM, Johansen KL, et al. Influence of race on kidney transplant outcomes within and outside the Department of Veterans Affairs. J Am Soc Nephrol 2005; 16: 269-77
17. Williams AF, Manias E, Gaskin CJ, Crawford L. Medicine non-adherence in kidney transplantation. J Ren Care 2014; 40: 107-116
18. Malek SK, Keys BJ, Kumar S, Milford E, Tullius SG. Racial and ethnic disparities in kidney transplantation. Transplant Int 2010; 24: 419-424
19. Office for National Statistics 2011 Census; KS201EW Ethnic group: local authorities in England and Wales. Accessed 2nd May 2016 at; www.ons.gov.uk/ons/guide-method/census/2011
20. New York: State and County QuickFacts. Accessed 10th May 2016 at; www.quickfacts.census.gov/qfd/states/36000.html
21. Gondos A, Dohler B, Brenner H, Opelz G. Kidney Graft Survival in Europe and the United States: Strikingly Different Long-Term Outcomes. Transplantation 2013; 95: 267-274
22. Young CJ, Gaston RS. Understanding the Influence of Ethnicity on Renal Allograft Survival. Am J Transplant 2005; 5: 2603-2604
23. Goldfarb-Rumyantzev AS, Koford JK, Baird BC, Chelamcharla M, Habib AN, Wang BJ, et al. Role of socioeconomic status in kidney transplant outcome. Clin J Am Soc Nephrol 2006; 1: 313–322
24. Petersen E, Baird BC, Barenbaum LL, Leviatov A, Koford JK, Shihab F, et al. The impact of employment status on recipient and renal allograft survival. Clin Transplant 2008; 22: 428–438
25. Krieger N. Theories for social epidemiology in the 21st Century: an ecosocial perspective. Int J Epidemiol 2001; 30: 668-877
26. Kucirka LM, Grams ME, Lessler J, et al. Association of Race and Age With Survival Among Patients Undergoing Dialysis. JAMA 2011; 306: 620-626
27. Smith SR, Butterly DW. Declining influence of race on the outcome of living-donor renal transplantation. Am J Transplant 2002; 2: 282–286

For peer review only

For peer review only - http://bmjopen.bmj.com/site/about/guidelines.xhtml

Table 1. Baseline demographics of Black kidney transplant recipients in England versus New York State between 2003 and 2013[1]

Total		England (n=1215)	NYS (n=2660)	P-value
**Age**	Mean ± standard deviation	46.3 ± 12.4	50.1 ± 13.5	<0.001*
**Total Length of Stay**	Median ± interquartile range	10 ± (IQR 8-16)	6 ± (IQR 5-9)	<0.001**
**Post transplant hospital stay**	Median ± interquartile range	8 ± (IQR 6-13)	6 ± (IQR 4-8)	<0.001**
**Sex**	Male	743 (61.2%)	1511 (56.8%)	0.011***
	Female	472 (38.8%)	1149 (43.2%)	
**Type of Donor**	Living	343 (28.2%)	548 (20.6%)	<0.001***
	Deceased	856 (70.5%)	1555 (58.5%)	
	Unknown	16 (1.3%)	557 (20.9%)	
**Medical co-morbidities at time of transplantation**	Diabetes	194 (16.0%)	741 (27.9%)	<0.001***
	Acute MI	25 (2.1%)	99 (3.7%)	
	Cerebrovascular disease	14 (1.2%)	46 (1.7%)	
	Congestive heart failure	22 (1.8%)	220 (8.3%)	
	Liver disease	6 (0.5%)	16 (0.6%)	
	Peptic ulcer	8 (0.7%)	31 (1.2%)	
	Peripheral vascular disease	13 (1.1%)	83 (3.1%)	

[1] * t-test, ** Mann-Whitney, *** chi-squared, NYS= New York State

For peer review only - http://bmjopen.bmj.com/site/about/guidelines.xhtml

Table 2. Comparing 1-year and follow up outcomes after kidney transplantation for Black patients in England versus New York State between 2003 and 2014[2]

Variable	England	NYS	p-value
1-year follow up			
**Emergency Readmission**	782 (64.36%)	892 (33.53%)	<0.001
**Cardiac Event**	16 (1.32%)	79 (2.97%)	0.002
**Stroke**	12 (0.99%)	23 (0.86%)	<0.001
**Cancer**	17 (1.40%)	26 (0.98%)	0.245
**Fracture**	5 (0.41%)	18 (0.68%)	0.319
**Transplant Rejection/Failure**	226 (18.6%)	702 (26.4%)	<0.001
Any time Post-Transplant			
**Number of Emergency Readmissions (mean)**	4.33	3.99	0.0697
**Cardiac Event**	72 (5.93%)	336 (12.63%)	<0.001
**Stroke**	47 (3.87%)	125 (4.70%)	0.224
**Cancer**	71 (5.84%)	171 (6.43%)	0.485
**Fracture**	36 (2.96%)	84 (3.16%)	0.745
**Transplant Rejection/Failure**	367 (30.2%)	1590 (59.8%)	<0.001
Mortality			
**In Hospital Death**	9 (0.74%)	36 (1.35%)	0.099
**30 day mortality**	12 (0.99%)	61 (2.29%)	0.006
**12 month mortality**	34 (2.80%)	173 (6.50%)	<0.001

[2] Obtained using chi-squared tests

For peer review only - http://bmjopen.bmj.com/site/about/guidelines.xhtml

Table 3. Adjusted Logistic regression analysis of kidney transplant mortality in both England and New York State by ethnicity between 2003 and 2014[3]

	30 Day mortality			
	ENGLAND		NYS	
**Ethnicity**	**Odds Ratio**	**p-value***	**Odds Ratio**	**p-value***
**Black**	1 (baseline group)		1 (baseline group)	
**Other**	1.48 (0.76,2.88)	0.246	0.79 (0.55,1.15)	0.214
**White**	0.81 (0.44,1.49)	0.491	0.62 (0.44,0.86)	0.005

	1 Year Mortality			
	ENGLAND		NYS	
**Ethnicity**	**Odds Ratio**	**p-value***	**Odds Ratio**	**p-value***
**Black**	1 (baseline group)		1 (baseline group)	
**Other**	1.27 (0.85,1.90)	0.234	0.79 (0.63, 0.99)	0.064
**White**	0.94 (0.65, 1.34)	0.728	0.66 (0.55,0.81)	<0.001

[3] Adjusted for age, sex, admission method and number of emergency readmissions.

For peer review only - http://bmjopen.bmj.com/site/about/guidelines.xhtml

For peer review only

Table 4. Adjusted Cox regression analysis for transplant rejection/failure among Black kidney transplant patients in England and NYS between 2003 and 2014.

		Hazard Ratio (95% CI)	p-value
**Age**		1.00 (0.99-1.00)	0.171
**Sex**	**Male**	1 (baseline group)	
	**Female**	1.00 (0.91,1.09)	0.928
**Type of Donor**	**Alive**	1 (baseline group)	
	**Dead**	1.37 (1.22,1.54)	<0.001
	**Unknown**	1.08 (0.92,1.27)	0.339
**Diabetes**		1.09 (0.98,1.20)	0.114
**Acute MI**		0.99 (0.84,1.17)	0.935
**CVF**		1.04 (0.87,1.24)	0.697
**PVD**		1.16 (0.98,1.37)	0.086
**CHF**		1.16 (1.04,1.30)	0.008
**Year**	**Pre 2007**	1 (baseline group)	
	**Post 2007**	1.30 (1.16,1.45)	<0.001
**Country**	**England**	1 (baseline group)	
	**NYS**	2.15 (1.91,2.43)	<0.001

For peer review only

For peer review only - http://bmjopen.bmj.com/site/about/guidelines.xhtml

Supplementary Table 1. Adjusted Cox regression analysis for transplant rejection/failure among White kidney transplant patients in England and NYS between 2003 and 2014.

		Hazard Ratio (95% CI)	p-value
**Age**		0.99 (0.99, 1.00)	<0.001
**Sex**	**Male**	1 (baseline group)	0.528
	**Female**	1.02 (0.97, 1.07)	
**Type of Donor**	**Alive**	1 (baseline group)	
	**Dead**	1.22 (1.26,1.40)	<0.001
	**Unknown**	0.99 (0.91,1.09)	0.878
**Diabetes**		1.17 (1.10, 1.24)	<0.001
**Acute MI**		1.10 (1.01, 1.20)	0.025
**CVF**		1.17 (1.06, 1.29)	0.002
**PVD**		1.14 (1.04, 1.25)	0.007
**CHF**		1.15 (1.06, 1.24)	0.001
**Year**	**Pre 2007**	1 (baseline group)	
	**Post 2007**	1.18 (1.12, 1.25)	<0.001
**Country**	**England**	1 (baseline group)	
	**NYS**	2.15 (2.04, 2.27)	<0.001

For peer review only

Figure legends

Figure 1. Unadjusted Kaplan Meier curve of kidney transplant rejection/failure for Black kidney transplant patients in England versus New York State between 2003 and 2014 (log-rank test p<0.001).

Supplementary Figure 1. Flowchart of patients excluded from analysis from both England and New York State cohorts

For peer review only

For peer review only - http://bmjopen.bmj.com/site/about/guidelines.xhtml

Figure 1. Cox regression plot of kidney transplant rejection/failure for Black kidney transplant patients in England versus New York State

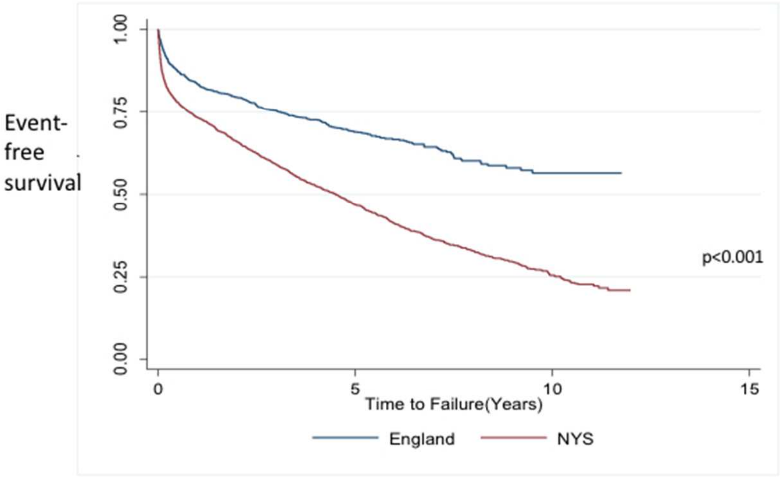

338x190mm (54 x 54 DPI)

review only

Supplementary Figure 1. Cohort study exclusions from England and New York State data

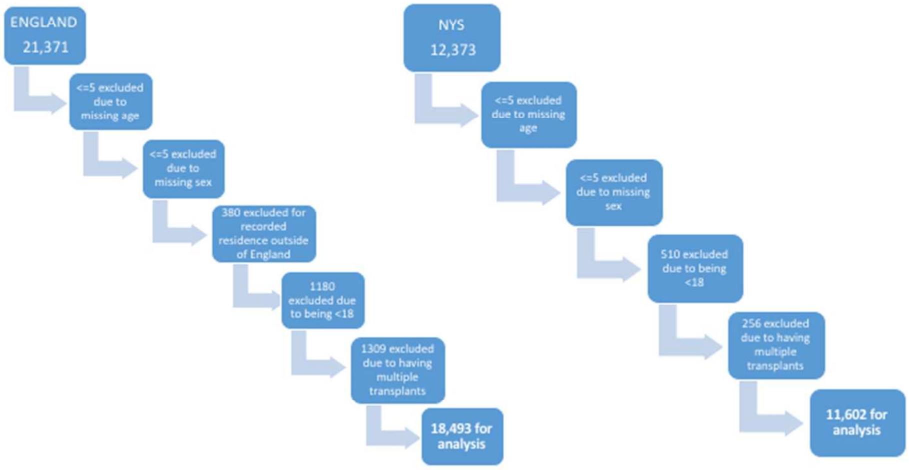

338x190mm (54 x 54 DPI)

review only

Supplementary Tables showing sensitivity analyses

		30 Day Mortality					
		England			NYS		
		Pre-Sensitivity Analysis	Black and Unknown combined	White and unknown combined	Pre-Sensitivity Analysis	Black and Unknown combined	White and unknown combined
Age Category	<50	1 (baseline group)	1 (baseline group)	1 (baseline group)	1 (baseline group)	1 (baseline)	1 (baseline)
	50+	3.76 (2.64,5.37)	3.76 (2.64,5.36)	3.76 (2.64,5.37)	1.21 (0.91,1.61)	1.21 (0.91,1.61)	1.21 (0.91,1.61)
Sex	Male	1 (baseline group)	1 (baseline group)	1 (baseline group)	1 (baseline group)	1 (baseline)	1 (baseline)
	Female	1.12 (0.82,1.54)	1.12 (0.82,1.54)	1.13 (0.82,1.55)	0.88 (0.66,1.17)	0.88 (0.66,1.17)	0.88 (0.66,1.17)
Admission Method	Elective	1 (baseline group)	1 (baseline group)	1 (baseline group)	1 (baseline group)	1 (baseline)	1 (baseline)
	Emergency	2.47 (1.75,3.50)	2.47 (1.74,3.49)	2.47 (1.75,3.49)	2.00 (1.49,2.68)	2.00 (1.49,2.68)	2.00 (1.49,2.68)
	Other	0.51 (0.07,3.79)	0.51 (0.07,3.73)	0.52 (0.07,3.86)	2.52 (0.59,10.71)	2.53 (0.60,10.74)	2.53 (0.60,10.75)
Number of Emergency Readmissions		0.20 (0.15,0.27)	0.20 (0.15,0.27)	0.20 (0.15,0.27)	0.77 (0.72,0.83)	0.77 (0.72,0.83)	0.77 (0.72,0.83)
Ethnic Group	Black	1 (baseline group)	1 (baseline group)	1 (baseline group)	1 (baseline group)	1 (baseline)	1 (baseline)
	Other	1.48 (0.76,2.88)	1.60 (0.97,2.63)	1.43 (0.73,2.82)	0.79 (0.55,1.15)	0.79 (0.54,1.14)	0.79 (0.54,1.15)
	White	0.81 (0.44,1.49)	0.79 (0.52,1.19)	0.73 (0.39,1.34)	0.62 (0.44,0.86)	0.61 (0.44,0.85)	0.62 (0.45,0.87)

		12 Month Mortality					
		England			NYS		
		Pre-Sensitivity Analysis	Black and Unknown combined	White and unknown combined	Pre-Sensitivity Analysis	Black and Unknown combined	White and unknown combined
Age Category	<50	1 (baseline group)	1 (baseline)	1 (baseline)	1 (baseline group)	1 (baseline)	1 (baseline)
	50+	3.97 (3.24,4.86)	3.94 (3.22,4.83)	3.97 (3.24,4.86)	1.45 (1.21,1.73)	1.21 (0.91,1.61)	1.21 (0.91,1.61)
Sex	Male	1 (baseline group)	1 (baseline)	1 (baseline)	1 (baseline group)	1 (baseline)	1 (baseline)
	Female	1.07 (0.89,1.27)	1.06 (0.89,1.27)	1.06 (0.89,1.27)	1.06 (0.89,1.26)	0.88 (0.66,1.17)	0.88 (0.66,1.17)
Admission Method	Elective	1 (baseline group)	1 (baseline)	1 (baseline)	1 (baseline group)	1 (baseline)	1 (baseline)
	Emergency	1.84 (1.53,2.22)	1.85 (1.54,2.23)	1.84 (1.52,2.22)	1.79 (1.50,2.13)	2.00 (1.49,2.68)	2.00 (1.49,2.68)
	Other	1.17 (0.59,2.33)	1.19 (0.60,2.37)	1.18 (0.59,2.33)	1.76 (0.62,4.98)	2.53 (0.60,10.74)	2.53 (0.60,10.75)
Number of Emergency Readmissions		0.84 (0.82,0.87)	0.84 (0.81,0.87)	0.84 (0.81,0.87)	0.89 (0.86,0.92)	0.77 (0.72,0.83)	0.77 (0.72,0.83)
Ethnic Group	Black	1 (baseline group)	1 (baseline)	1 (baseline)	1 (baseline group)	1 (baseline)	1 (baseline)
	Other	1.24 (0.85,1.90)	1.60 (1.17,2.18)	1.27 (0.84,1.91)	0.79 (0.63,0.99)	0.79 (0.54,1.14)	0.79 (0.54,1.15)
	White	0.94 (0.65,1.34)	1.19 (0.92,1.54)	0.91 (0.63,1.32)	0.66 (0.55,0.81)	0.61 (0.44,0.85)	0.62 (0.45,0.87)

Living and Unknown Combined

		Hazard Ratio (95% CI)	p-value
Age		1.00 (0.99,1.00)	0.181
Sex	Male	1 (baseline group)	
	Female	0.99 (0.91,1.09)	0.897
Type of Donor	Alive/unknown	1 (baseline group)	
	Dead	1.33 (1.20,1.46)	<0.001
Diabetes		1.08 (0.98,1.20)	0.12
Acute MI		0.99 (0.85,1.17)	0.945
CVF		1.03 (0.87,1.23)	0.716
PVD		1.16 (0.98,1.38)	0.086
CHF		1.16 (1.04,1.30)	0.008
Year	Pre 2007	1 (baseline group)	
	Post 2007	1.27 (1.15,1.41)	<0.001
Country	England	1 (baseline group)	
	NYS	2.17 (1.93,2.45)	<0.001

Deceased and Unknown Combined

		Hazard Ratio (95% CI)	p-value
Age		1.00 (0.99,1.00)	0.24
Sex	Male	1 (baseline group)	
	Female	1.00 (0.91,1.09)	0.96
Type of Donor	Alive	1 (baseline group)	
	Dead/Unknown	1.31 (1.16,1.47)	<0.001
Diabetes		1.09 (0.98,1.20)	0.103
Acute MI		0.99 (0.85,1.17)	0.945
CVF		1.04 (0.87,1.24)	0.655
PVD		1.17 (0.98,1.38)	0.077
CHF		1.18 (1.06,1.32)	0.004
Year	Pre 2007	1 (baseline group)	
	Post 2007	1.41 (1.27,1.56)	<0.001
Country	England	1 (baseline group)	
	NYS	2.06 (1.83,2.32)	<0.001

Alive donors only

		Hazard Ratio (95% CI)	p-value
Age		0.98 (0.97,0.99)	<0.001
Sex	Male	1 (baseline group)	
	Female	1.13 (0.92,1.39)	0.248
Diabetes		1.25 (0.98,1.58)	0.071
Acute MI		1.12 (0.74,1.70)	0.596
CVF		0.64 (0.41,1.02)	0.058
PVD		1.56 (1.05,2.31)	0.028
CHF		1.29 (0.99,1.69)	0.058
Year	Pre 2007	1 (baseline group)	
	Post 2007	1.34 (1.05,1.72)	0.02
Country	England	1 (baseline group)	
	NYS	1.68 (1.32,2.13)	<0.001

Deceased donors only

		Hazard Ratio (95% CI)	p-value
Age		1.00 (1.00,1.00)	0.915
Sex	Male	1 (baseline group)	
	Female	1.00 (0.90,1.13)	0.934
Diabetes		1.10 (0.97,1.25)	0.144
Acute MI		0.96 (0.79,1.16)	0.645
CVF		1.15 (0.94,1.41)	0.178
PVD		1.07 (0.87,1.31)	0.515
CHF		1.13 (0.99,1.30)	0.075
Year	Pre 2007	1 (baseline group)	
	Post 2007	1.25 (1.09,1.43)	0.001
Country	England	1 (baseline group)	
	NYS	2.25 (1.95,2.59)	<0.001

For peer review only

Supplementary Table 1. Adjusted Cox regression analysis for transplant rejection/failure among White kidney transplant patients in England and NYS between 2003 and 2014.

		Hazard Ratio (95% CI)	p-value
**Age**		0.99 (0.99, 1.00)	<0.001
**Sex**	**Male**	1 (baseline group)	0.528
	**Female**	1.02 (0.97, 1.07)	
**Type of Donor**	**Alive**	1 (baseline group)	
	**Dead**	1.22 (1.26,1.40)	<0.001
	**Unknown**	0.99 (0.91,1.09)	0.878
**Diabetes**		1.17 (1.10, 1.24)	<0.001
**Acute MI**		1.10 (1.01, 1.20)	0.025
**CVF**		1.17 (1.06, 1.29)	0.002
**PVD**		1.14 (1.04, 1.25)	0.007
**CHF**		1.15 (1.06, 1.24)	0.001
**Year**	**Pre 2007**	1 (baseline group)	
	**Post 2007**	1.18 (1.12, 1.25)	<0.001
**Country**	**England**	1 (baseline group)	
	**NYS**	2.15 (2.04, 2.27)	<0.001

For peer review only
